# Engineering of *Yarrowia lipolytica* for the production of plant triterpenoids: Asiatic, madecassic, and arjunolic acids

**DOI:** 10.1016/j.mec.2022.e00197

**Published:** 2022-03-26

**Authors:** Jonathan Asmund Arnesen, Arian Belmonte Del Ama, Sidharth Jayachandran, Jonathan Dahlin, Daniela Rago, Aaron John Christian Andersen, Irina Borodina

**Affiliations:** a The Novo Nordisk Foundation Center for Biosustainability, Technical University of Denmark, Kemitorvet 220, 2800, Kgs. Lyngby, Denmark; b Department of Biotechnology and Biomedicine, Technical University of Denmark, Søltofts plads 221, 2800, Kgs. Lyngby, Denmark

**Keywords:** Metabolic engineering, Terpenoid, Natural products, Yeast, Yarrowia lipolytica, Synthetic biology

## Abstract

Several plant triterpenoids have valuable pharmaceutical properties, but their production and usage is limited since extraction from plants can burden natural resources, and result in low yields and purity. Here, we engineered oleaginous yeast *Yarrowia lipolytica* to produce three valuable plant triterpenoids (asiatic, madecassic, and arjunolic acids) by fermentation. First, we established the recombinant production of precursors, ursolic and oleanolic acids, by expressing plant enzymes in free or fused versions in a *Y. lipolytica* strain previously optimized for squalene production. Engineered strains produced up to 11.6 mg/g DCW ursolic acid or 10.2 mg/g DCW oleanolic acid. The biosynthetic pathway from ursolic acid was extended by expressing the *Centella asiatica* cytochrome P450 monoxygenases CaCYP716C11p, CaCYP714E19p, and CaCYP716E41p, resulting in the production of trace amounts of asiatic acid and 0.12 mg/g DCW madecassic acid. Expressing the same *C. asiatica* cytochromes P450 in oleanolic acid-producing strain resulted in the production of oleanane triterpenoids. Expression of CaCYP716C11p in the oleanolic acid-producing strain yielded 8.9 mg/g DCW maslinic acid. Further expression of a codon-optimized CaCYP714E19p resulted in 4.4 mg/g DCW arjunolic acid. Lastly, arjunolic acid production was increased to 9.1 mg/g DCW by swapping the N-terminal domain of CaCYP714E19p with the N-terminal domain from a *Kalopanax septemlobus* cytochrome P450. In summary, we have demonstrated the production of asiatic, madecassic, and arjunolic acids in a microbial cell factory. The strains and fermentation processes need to be further improved before the production of these molecules by fermentation can be industrialized.

## Highlights

• Engineered *Yarrowia lipolytica* strains to produce ursane and oleanane triterpenoids.
• Among ursanes are ursolic, asiatic, and madecassic acids*.*
• Among oleananes are oleanolic, maslinic, arjunolic acids, and hederagenin.
• Fusing β-amyrin synthase and squalene epoxidase improved oleanolic acid production.
• *Y. lipolytica* is a promising host for complex plant triterpenoid production.

## 1 Introduction

**Fig. 1:**
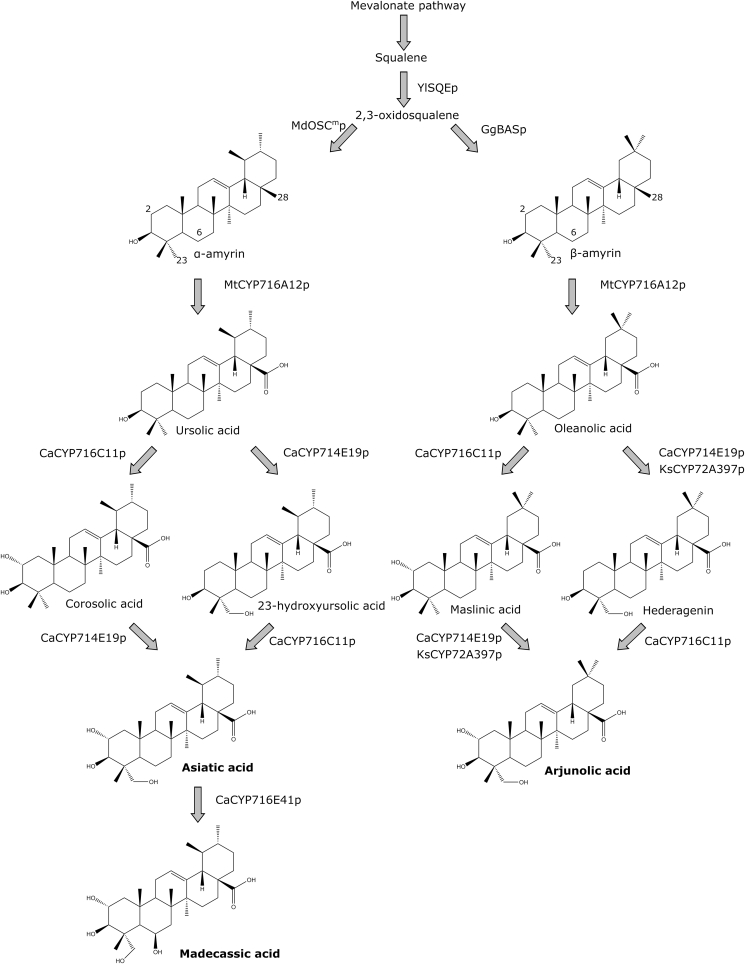
Overview of the heterologous pathways constructed in yeast. YlSQEp, Yarrowia lipolytica squalene epoxidase. MdOSC1^m^p, mutated Malus x Domestica oxidosqualene cyclase. GgBASp, Glycyrrhiza Glabra β-amyrin synthase. CaCYP, Centella asiatica cytochrome P450. KsCYP, Kalopanax semtemlobus cytochrome P450. Modified carbon positions 2, 6, 23, and 28 are enumerated.

Some natural plant triterpenoids possess anticancer, immunomodulatory, or antimicrobial effects (Bishayee et al., 2011; Ríos, 2010). Extraction from plants can be complicated due to low content of the triterpenoids or due to co-extraction of impurities, while straining natural resources (Englund et al., 2015; Idris and Mohd Nadzir, 2021; Mora-Pale et al., 2014). Furthermore, the application or ingestion of plant extracts may cause adverse effects in some people (Gomes et al., 2010; Jorge and Jorge, 2005). An alternative method for manufacturing these compounds is by fermentation of engineered microbes that express the specific plant biosynthetic enzymes. Triterpenoids are produced mainly through the mevalonate pathway, which converts acetyl-CoA to the phosphorylated carbon 5 (C_5_)-units IPP and DMAPP (Liao et al., 2016). The condensation of IPP and DMAPP molecules leads to the formation of C_15_ farnesyl diphosphate, which can be enzymatically condensated and dephosphorylated to form C_30_ squalene. Squalene is converted by squalene epoxidases (SQEp) into 2,3-oxidosqualene, a common precursor of sterols and triterpenoids. Specialized triterpenoids produced by plants may confer resistance to predators or inhibit the growth of competing plants (González-Coloma et al., 2011; Wang et al., 2014). Many triterpenoids also have beneficial effects on humans and animals. For example, the aglycones asiatic and madecassic acid and their glycosylated derivatives asiaticoside and madecassicoside are commonly found in extracts of *Centella asiatica* (gotu kola), which are widely used in cosmetics (Bylka et al., 2013). The extracts of *C. asiatica* can positively affect conditions like diabetes, obesity, neurological and cardiovascular diseases, and skin wounds, and the effects are often attributed to the beforementioned triterpenoids (Sun et al., 2020). Asiatic and madecassic acid are formed by cyclization of 2,3-oxidosqualene into α-amyrin, which is probably then carboxy- and hydroxylated by cytochromes P450 (Fig. 1) (Andre et al., 2016; Kim et al., 2018; Miettinen et al., 2017). Likewise, β-amyrin is formed by cyclization of 2,3-oxidosqualene by terpenoid synthases (Hayashi et al., 2001). Several rounds of oxygenation by cytochromes P450 can convert β-amyrin into hederagenin, maslinic acid, and arjunolic acid, which all possess useful properties (Kim et al., 2018). Arjunolic acid, found in the arjun tree *Terminalia arjuna*, has antidiabetic, cardioprotective, and anti-inflammatory properties (Ghosh and Sil, 2013; Hemalatha et al., 2010; Ramesh et al., 2012).

We aimed to produce valuable α- and β-amyrin-derived triterpenoids in the oleaginous yeast *Yarrowia lipolytica*. This yeast has an inherently high acetyl-CoA flux, a sequenced genome, a comprehensive toolkit available for genetic modification, and a safe history of use, where several *Y. lipolytica* strains have received GRAS-status (Christen and Sauer, 2011; Dujon et al., 2004; Holkenbrink et al., 2018; Turck et al., 2019). Furthermore, *Y. lipolytica* accumulates high amounts of lipids, which can be useful for storing hydrophobic triterpenoids (Beopoulos et al., 2009). Moreover, *Y. lipolytica* has already been used to produce many terpenoids like astaxanthin, betulinic acid, oleanolic acid, and gibberellin phytohormones (Jin et al., 2019; Kildegaard et al., 2017, 2021; Li et al., 2020). Therefore, we sought to leverage the benefits of *Y. lipolytica* and modern engineering tools to construct triterpenoid yeast cell factories.

## 2 Methods

### 2.1 Strains and media

The strains used in this study are listed in Supplementary Table S1. A pre-engineered *Y. lipolytica* strain with a high flux towards 2,3-oxidosqualene biosynthesis was used to construct the strains in this study (Arnesen et al., 2020). The strain was previously constructed from the W29-derived strain ST6512 (*MATa ku70Δ::PrTEF1-Cas9-TTef12::PrGPD-DsdA-TLip2*) that expressed Cas9p for CRISPR/based DNA integration (Holkenbrink et al., 2018; Marella et al., 2020). In turn, ST6512 was based on the *Y. lipolytica* strain Y-63746 (*MATa*), a kind gift from the ARS Culture Collection, NCAUR, USA. Plasmid construction was done with the *Escherichia coli* DH5α strain that was cultivated on lysogeny broth (LB) media with 100 mg/L ampicillin at 37 °C and 300 rpm shaking. The *Y. lipolytica* cells were cultivated at 30 °C on media containing 10 g/L yeast extract, 20 g/L peptone, and 20 g/L glucose (YPD) with 20 g/L agar added for solid media. Hygromycin (400 mg/L) or nourseothricin (250 mg/L) was added to the media for yeast cell selection. For yeast cultivations, YPD-media with 80 g/L glucose (YPD80) instead of 20 g/L was used. All chemicals were purchased from Sigma-Aldrich, unless otherwise noted. Nourseothricin was obtained from Jena BioScience GmbH (Germany).

### 2.2 Plasmids

The Supplementary Tables S2, S4, and S5 list the plasmids, biobricks, and primers, respectively, used in this study. Primer3 was used to design some primers (Untergasser et al., 2012). The Biobricks were PCR-amplified with Phusion U Polymerase (Thermo Scientific), and USER cloning was used to assemble the EasyCloneYALI plasmids that were transformed into *E. coli* (Holkenbrink et al., 2018). Correct plasmid assembly was verified by sequencing. The synthetic genes encoding *GgBAS* (Genbank: Q9MB42.1), *MdOSC1*^*m*^ (genbank: ACM89977.1) with the following mutations N11T/P250H/P373A based on (Yu et al., 2020), *MtCYP716A12* (genbank: CBN88268.1), *KsCYP72A397* (genbank: ALO23113.1), *CaCYP716C11 (*genbank: AOG74835.1), *CaCYP714E19 (*genbank mRNA sequence: KT004520.1), and *CaCYP716E41* (genbank: AOG74834.1) were ordered from Thermo Fischer Scientific. The genes were codon-optimized for *Y. lipolytica,* and the algorithms used for codon-optimization can be seen in Supplementary Table S3. The DNA sequences of the synthetic genes can be found in the supplementary materials. For some of the genes, we used a codon-optimization algorithm based on codon usage of highly expressed genes (*IhOP*). The algorithm uses a custom codon usage table calculated from the 100 most highly expressed genes in the previously published *Y. lipolytica* RNA-seq dataset (Dahlin et al., 2019). The algorithm was designed to preferentially select the most abundant codon for a given amino acid, and to allow for the selection of alternative codons to work around sequence constraints, such as avoiding restriction sites, polynucleotide stretches, and high or low GC content. The *IhOP*-algorithm and the codon usage table are available via github (https://github.com/CfB-YME/YALI_opt). The sequence of *AtATR2* was described in a previous study (Kildegaard et al., 2021). The Emboss needle global pairwise sequence alignment algorithm was used to align nucleotide sequences (Madeira et al., 2019).

### 2.3 Strain construction

Supplementary Table S1 lists the yeast strains used in this study. The integrative plasmids were *Not*I-digested prior to transformation, which was based on a previously described lithium acetate protocol (Holkenbrink et al., 2018). Colony PCR with primers complementary to the plasmid and adjacent genomic region was used to confirm genomic integration.

### 2.4 Cultivation and metabolite extraction

Precultures were made by inoculating 2.5 mL YPD media in 24-deep-well plates with an air-penetrable lid (EnzyScreen, NL) with a yeast colony and growing the cultures overnight at 30 °C with shaking at 300 rpm. Then, 2.5 mL of YPD80 was inoculated from the precultures to an OD600 of 0.1 and the cultures were left to grow at 30 °C with 300 rpm shaking for 72 h. By the end of cultivation, dry cell weight (DCW) was measured by transferring 1 mL culture broth to a pre-weighed 2 mL microcentrifuge tube (Sarstedt), which was centrifuged, and the supernatant was removed. The remaining cell pellets were then dried at 60 °C for a minimum of 72 h and weighted.

For metabolite extraction, 1 mL of culture broth was transferred to a 2 mL microcentrifuge tube, which was centrifuged, and the supernatant was removed. The cell pellets were washed twice with water before 500 μL of 0.212–0.3 mm acid-washed beads, and 1 mL of 99% ethanol was added to the cell pellet. The tubes were then bead-bashed at 6500 rpm three times for 45 s with 15 s pause on a Precellys®24 homogenizer (Bertin Corp.). The tubes were then centrifuged, and the ethanolic supernatant was sampled for analysis.

### Analytical methods

For detection and quantification of ursolic acid, asiatic acid, and madecassic acid, 200 μL ethanolic sample extract and 10 μL of internal standard (cholesterol dissolved in ethanol) were combined and evaporated. 50 μL N*,O*-Bis(trimethylsilyl)trifluoroacetamide (BSTFA) and 50 μL pyridine were added to the dried samples, which were then incubated at 80 °C for 30 min, shaken at 650 rpm in a tabletop heatblock. After derivatization, 900 μL hexane was added for quantification of ursolic acid and qualitative analysis of asiatic and madecassic acid. No Hexane was added for quantification of madecassic acid.

Quantitative and qualitative analysis of ursolic, asiatic, and madecassic acid was performed with a Thermo Scientific™ ISQ Series single quadrupole GC-MS system with a Thermo Scientific™ TRACE™ 1300 GC system equipped with the Thermo Scientific™ Instant Connect Split/Splitless (SSL) Injector and a CTC Analytical CombiPAL Autosampler. For quantification of ursolic acid, 1 μL of the hexane-diluted sample was injected into a Thermo Scientific™ TraceGOLD TG-5MS column (30 m × 0.25 mm × 0.25 μm) in splitless mode with a constant helium flow of 1.5 mL/min. For quantification of madecassic acid, 1 μL of undiluted samples was injected, and for qualitative analysis of asiatic and madecassic acid 10 μL of hexane diluted samples were injected. The injector was set to 300 °C, and the oven was set to 80 °C which was held for 1.5 min post-injection. Then, the oven temperature ramped to 300 °C at 20 °C/min, and the temperature was held for 25.5 min resulting in a total run time of 38 min. The MS transfer line was set to 250 °C and the MS ion source at 300 °C. The acquisition mode was Full-scan with the m/z range of 50–800 with a solvent delay of 4 min. The ion source was ExtractaBrite (Thermo Fisher Scientific), the EI ionization was 70 eV, and the acquisition rate was 0.2 s. The data was processed with the Chromeleon 7.2.9 and 7.2.10 software (Thermo Fisher Scientific), and the compound concentrations were calculated from authentic calibration standards.

Oleanolic acid concentrations were quantified using a Dionex 3000 HPLC system connected to an Orbitrap Fusion Mass Spectrometer (Thermo Fisher Scientific, San Jose, CA). This method was also used to confirm the identity of arjunolic and maslinic acids, and hederagenin in our samples. The chromatographic separation was achieved using a Waters ACQUITY BEH C18 (10 cm × 2.1 mm, 1.7 μm) column equipped with an ACQUITY BEH C18 guard column kept at 40 °C with a flow rate of 0.35 mL/min. The mobile phases consisted of MilliQ© water +0.1% formic acid (A) and acetonitrile + 0.1% formic acid (B). The initial composition was 2% B, held for 0.8 min, followed by a linear gradient to 5% B over 3.3 min, and afterward, 100% B was reached over 10 min and held for 2 min before going back to initial conditions. Re-equilibration time was 2.7 min. The injection volume was set at 1 μL and underivatized, ethanolic cell extracts were injected. The MS(MS) measurement was done in positive-heated electrospray ionization (HESI) mode with a voltage of 2500 V acquiring in full MS/MS spectra (Data Dependent Acquisition-driven MS/MS) with a m/z range of 70–1000. The MS1 resolution was set at 120,000 and at 30,000 for the MS2. Precursor ions were fragmented by stepped Higher-Energy C-trap dissociation (HCD) using collision energies of 20, 40, and 55 eV. Authentic standards were used to quantify oleanolic acid concentrations and to confirm the identity of arjunolic and maslinic acids, and hederagenin in our samples.

Hederagenin, maslinic acid, and arjunolic acid concentrations were quantified with a Dionex 3000 HPLC system coupled to a diode array detector. To achieve separation, 10 μL underivatized, ethanolic sample was injected into an Agilent Zorbax Eclipse Plus C18 4.6 × 100 mm, 3.5 μm column (Agilent Technologies, Santa Clara, CA, USA) heated to 30 °C. The mobile phase consisted of a 0.05% acetic acid (A) and acetonitrile (B). The gradient started as 5% B and followed a linear gradient to 95% B over 8 min. This solvent composition was held for 2 min, after which it was changed immediately to 5% B and held until 12 min. The elution of the compounds was detected at a wavelength of 210 nm. HPLC data were processed using Chromeleon 7.2.9 software, and compound concentrations were calculated from authentic calibration standards.

## 3 Results

### 3.1 Production of ursolic and oleanolic acids

**Fig. 2:**
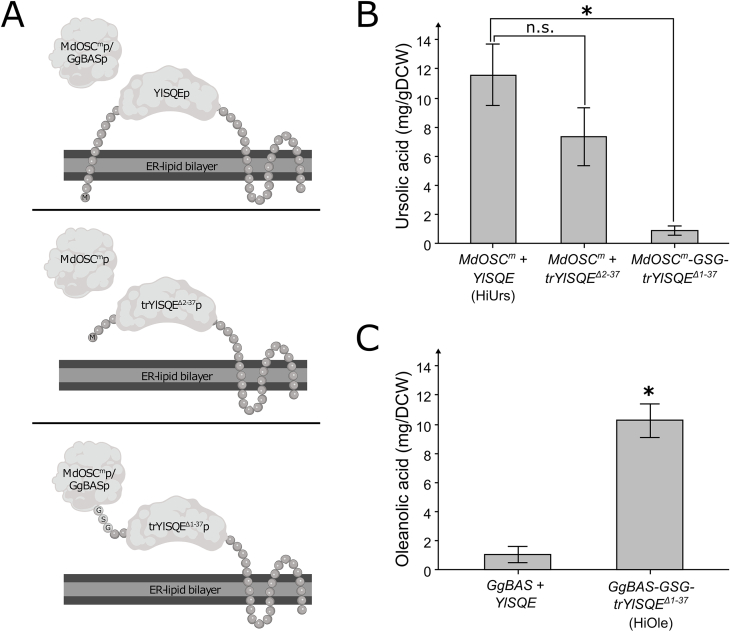
Production of ursolic and oleanolic acid in Y. lipolytica. **(A)** Overview of predicted protein structures of MdOSC^m^p, GgBASp, YlSQEp, and their modified versions. **(B)** Ursolic acid production by expression of MdOSC^m^ and YlSQE, MdOSC^m^ and TrYlSQE^Δ2-37^, or fusion construct MdOSC^m^-GSG- TrYlSQE^Δ1-37^ in a platform strain optimized for triterpenoid production expressing MtCYP716A12 and AtATR2. **(C)** Oleanolic acid production by expression of GgBAS and YlSQE, or fusion construct GgBAS-GSG- TrYlSQE^Δ1-37^ in a platform strain optimized for triterpenoid production expressing MtCYP716A12 and AtATR2. Averages and standard deviations are based on triplicate cultivations 24-deep well plates with YPD80-media. Asterisks indicate statistical significant difference compared to the expression of GgBAS and YlSQE (*t*-test, critical two-tailed, p < 0.05). n.s. not significant.

As the basic strain, we used a *Y. lipolytica* previously engineered for high flux towards 2,3-oxidosqualene (C_30_-platform strain). The C_30_-platform was previously modified by overexpression of the native ATP citrate lyase 1 (*YlACL*), 3-hydroxy-3-methylglutaryl coenzyme A reductase (*YlHMG*)*,* mevalonate kinase (*YlERG12*), isopentyl diphosphate isomerase (*YlIDI*), farnesyl diphosphate synthase (*YlERG20*), squalene synthase (*YlSQS*), and squalene epoxidase (*YlSQE*), expression of a *Salmonella enterica* acetyl-CoA synthetase (*SeACS*), and downregulation of the native lanosterol synthase (Arnesen et al., 2020). We then expressed the *Medicago truncatula* cytochrome P450 *MtCYP716A12* and *Arabidopsis thaliana* cytochrome P450 reductase *AtATR2* in the C_30_-platform strain (C_30,COOH_-platform strain). MtCYP716A12p can catalyze C-28 carboxylation of triterpenoids α-amyrin and β-amyrin, converting them into ursolic or oleanolic acid, respectively (Fig. 1) (Carelli et al., 2011; Fukushima et al., 2013; Suzuki et al., 2018). We then expressed a mutated version of *Malus x Domestica* oxidosqualene cyclase *MdOSC1*^*N11T/P250H/P373A*^ (*MdOSC1*^*m*^) to produce α-amyrin from 2,3-oxidosqualene (Yu et al., 2020). We also expressed an additional copy of *YlSQE* to enhance the convertion of squalene into 2,3-oxidosqualene because the C_30_-platform strain was shown to accumulate high squalene levels (Arnesen et al., 2020). We also expressed a fusion protein of MdOSC1^m^p linked to YlSQEp since linking enzymes may improve enzymatic activity (Guo et al., 2017; Hu et al., 2020). According to InterProScan, YlSQEp contains an N-terminal transmembrane (TM) domain localized inside the ER (Fig. 2A) (Jones et al., 2014). Therefore, the first 37 amino acids were deleted to generate trYlSQE^Δ1-37^p lacking the N-terminal transmembrane domain. A fusion protein of MdOSC^m^p C-terminally fused with a Gly-Ser-Gly (GSG)-linker to the N-terminal of trYlSQE^Δ1-37^p (MdOSC^m^-GSG-trYlSQE^Δ1-37^p) was expressed in the C_30,COOH_-platform strain. Additionally, MdOSC^m^p and trYlSQE^Δ2-37^p, with the initial methionine preserved, were also co-expressed in the C_30,COOH_-platform strain as a control. Co-expressing MdOSC^m^p with YlSQEp produced the highest ursolic acid titer of 254.3 ± 89.5 mg/L or 11.6 ± 2.1 mg/g DCW, and the strain was named HiUrs (Fig. 2B). Neither expression of the fusion protein MdOSC^m^-GSG-trYlSQE^Δ1-37^p nor co-expression of MdOSC^m^p and trYlSQE^Δ2-37^p proved comparatively beneficial for ursolic acid production.

We also constructed an oleanolic acid-producing strain by expressing a *Glycyrrhiza glabra* β-amyrin synthase (GgBASp) together with MtCYP716A12p and AtATR2p in the C_30_-platform strain (Hayashi et al., 2001). GgBASp was co-expressed with YlSQEp individually or as a fusion protein (GgBAS-GSG-trYlSQE^Δ1-37^p). Interestingly, the strain co-expressing GgBASp and YlSQEp only produced 28.6 ± 19.3 mg/L or 1.0 ± 0.6 mg/g DCW oleanolic acid, while the strain expressing GgBAS-GSG-trYlSQE^Δ1-37^p fusion (HiOle) produced 260.0 ± 50.1 mg/L or 10.2 ± 1.1 mg/g DCW oleanolic acid (*p* < 0.05, biomass specific yield, *t*-test, critical two-tailed, Fig. 2C).

### 3.2 Production of asiatic and madecassic acids

**Fig. 3:**
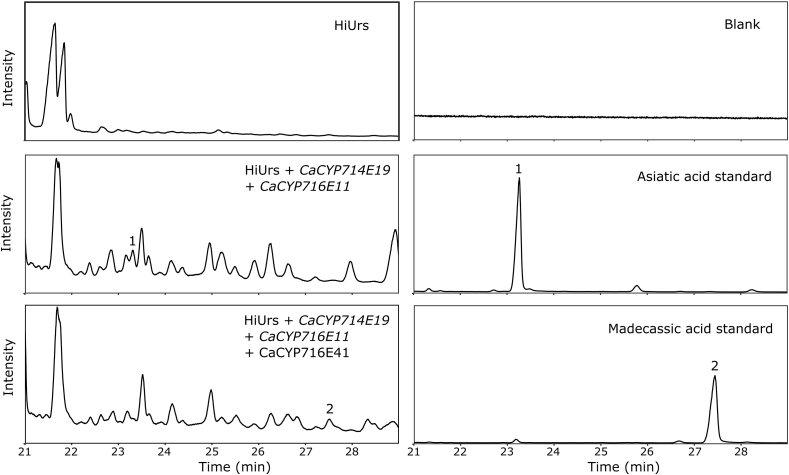
GC-MS total ion chromatograms of derivatized yeast samples and authentic triterpenoid standards. Numbers indicate the annotated triterpenoid peaks; 1, Asiatic acid. 2, Madecassic acid.

We then extended the biosynthetic pathway from ursolic acid to asiatic acid. *Centella asiatica* cytochromes P450 CaCYP716C11p and CaCYP714E19p can catalyze C-2 and C-23 hydroxylation of ursolic acid, respectively, to produce asiatic acid (Fig. 1) (Kim et al., 2018; Miettinen et al., 2017). Therefore, *CaCYP716C11* and *CaCYP714E19* were expressed in the HiUrs-strain. GC-MS analysis of cell extracts confirmed the production of asiatic acid based on retention time and MS spectral comparison of the sample peak with an authentic standard (Fig. 3 and Fig. S1). We could not calculate asiatic acid concentration due to the presence of co-eluting compounds, which were tentatively identified as triterpenoid-type products based on their MS spectra (data not shown). MdOSC1p is known to catalyze the formation of lupeol and β-amyrin, which also may be hydroxylated by CaCYP716C11p and CaCYP714E19p (Andre et al., 2016). Therefore, it is possible that oxygenated lupane and oleanane side products were produced as well. Next, we extended the pathway towards madecassic acid by additional expression of CaCYP716E41p, which can catalyze C-6 hydroxylation of asiatic acid (Miettinen et al., 2017). We were able to confirm the production of madecassic acid in the strain expressing *CaCYP716C11*, *CaCYP714E19*, and *CaCYP716E4*1 by GC-MS analysis, based on comparison of the retention time and MS spectrum of the sample and reference standard (Fig. 3 and Fig. S1). The production could be quantified at 3.1 ± 0.03 mg/L or 0.12 ± 0.005 mg/g DCW madecassic acid in the strain since no tentative triterpenoid-type compounds co-eluted with madecassic acid. To further demonstrate the potential of *Y. lipolytica* for the production of valuable triterpenoids, we decided to also construct oleanane triterpenoid cell factories, since the same cytochromes P450 can oxygenate oleanane triterpenoid backbones.

### 3.3 Production of arjunolic acid

**Fig. 4:**
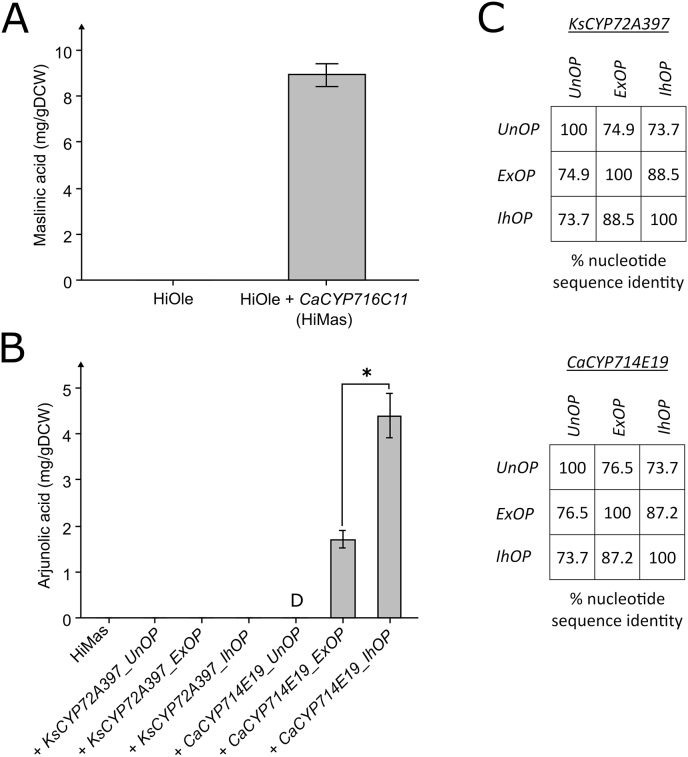
Production of maslinic and arjunolic acid in Y. lipolytica. **(A)** Maslinic acid production by expression of CaCYP716C11 in the oleanolic acid-producing strain HiOle. **(B)** Arjunolic acid production by expression of different codon-optimized versions of KsCYP72A397 and CaCYP716C11 in HiMas. UnOP, Unoptimized nucleotide sequence from native host. ExOP, nucleotide sequence codon-optimized with an algorithm from an external source. IhOP, nucleotide sequence codon-optimized with an algorithm based on codon usage of highly expressed genes. **(C)** Nucleotide sequence identities between the cytochrome P450 genes based on Needleman-Wunsch global alignment. Averages and standard deviations are based on triplicate cultivations 24-deep well plates with YPD80-media. Asterisks indicate statistical significant differences (*t*-test, critical two-tailed, p < 0.05). D, detected.

To extend the pathway from oleanolic acid to other valuable oleananes, we expressed CaCYP716C11p in the HiOle strain. CaCYP716C11p can catalyze the formation of maslinic acid by hydroxylating the C-2 position of oleanolic acid (Miettinen et al., 2017). The new strain (HiMas) produced 162.0 ± 10.0 mg/L or 8.9 ± 0.5 mg/g DCW maslinic acid (Fig. 4A). The presence of maslinic acid in HiMas was confirmed by LC-MS analysis with authentic standard comparison (Fig. S2). We then attempted to extend the pathway towards arjunolic acid, which necessitated the C-23 hydroxylation of maslinic acid. However, while CaCYP714E19p can catalyze the C-23 hydroxylation on oleanane triterpenoids, it can also generate a carboxyl group at the same position. Since the leakage of intermediates into C-23 carboxylated side-products would be undesirable, we attempted to express a cytochrome P450 from *Kalopanax septemlobus* (KsCYP72A397p) in the HiMas strain. KsCYP72A397p was shown only to produce the C-23 hydroxyl group, which could lead to a more efficient production of arjunolic acid (Han et al., 2018). Surprisingly, the expression of *KsCYP72A397* in the HiMas strain did not result in arjunolic acid production (Fig. 4B). Of note, the expressed version of *KsCYP72A397* was codon-optimized for *Y. lipolytica* by an algorithm from an external source (ExOP) (Swainston et al., 2014). The previous report showed that expressing the native *KsCYP72A397* sequence cloned from *K. septemlobus* cDNA in an oleanolic acid-producing *S. cerevisiae* strain resulted in the C-23 hydroxylation of oleanolic acid into hederagenin (Han et al., 2018). Therefore, we expressed the native sequence (*KsCYP72A397_UnOP*) that only shared 74.9% nucleotide sequence identity with *KsCYP72A397_ExOP*. We also tried to optimize the sequence with an in-house developed codon-optimization algorithm based on codon usage of highly expressed genes (*KsCYP72A397_IhOP*). The IhOP-algorithm applies a strict codon usage, based on the codon usage of the 100 highest expressed genes from *Y. lipolytica* (Dahlin et al., 2019). Using highly favored codons could potentially improve the expression of heterologous genes such as *KsCYP72A397*. Neither the expression of *KsCYP72A397_UnOP* nor *KsCYP72A397_IhOP* in HiMas resulted in arjunolic acid production (Fig. 4B). We then attempted to extend the pathway from maslinic acid to arjunolic acid by expression of *CaCYP714E19*.

To discern the effect of codon-optimization of *CaCYP714E19* for arjunolic acid production, we expressed *CaCYP714E19_UnOP, CaCYP714E19_*IhOP, which was also used for asiatic and madecassic acid production, or *CaCYP714E19_ExOP*, which was optimized by another external algorithm (Fath et al., 2011), in HiMas. The expression of *CaCYP714E19_UnOP* in HiMas resulted in detectable amounts of arjunolic acid (>1 mg/L) (Fig. 4B). While expression of *CaCYP714E19_ExOP* in HiMas resulted in 29.1 ± 5.5 mg/L and 1.7 ± 0.2 mg/g DCW arjunolic acid, the expression of *CaCYP714E19_IhOP* lead to 75.6 ± 18.8 mg/L and 4.4 ± 0.5 mg/g DCW arjunolic acid (biomass specific yield, *p* < 0.05). The identity of arjunolic acid was confirmed by LC-MS analysis with an authentic standard (Fig. S3). Interestingly, all maslinic acid was converted in the *CaCYP714E19_ExOP* and *CaCYP714E19_IhOP* expressing strains, while small amounts of hederagenin could be detected for these strains (Figs. S4 and S5). Furthermore, expression of the *KsCYP72A397*-and *CaCYP714E19*-codon variants in HiOle led to similar production of hederagenin; no production for the expression of any *KsCYP72A397*-codon variant, while the expression of all *CaCYP714E19*-codon variants led to hederagenin production (Figs. S6 and S7).

**Fig. 5:**
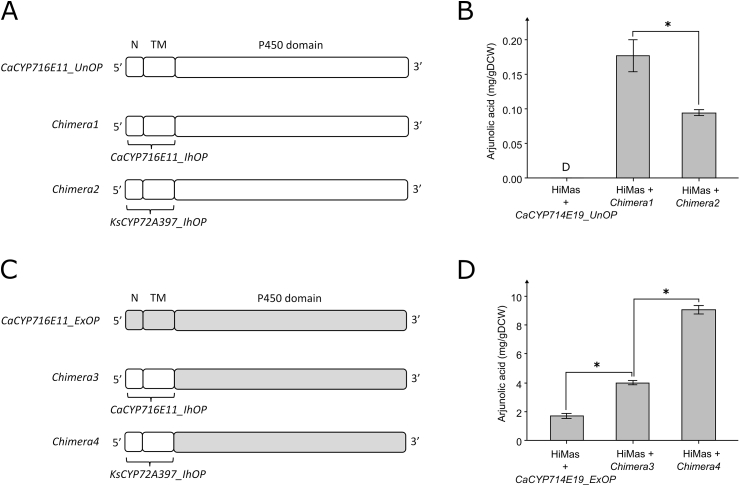
Arjunolic acid production in Y. lipolytica by expression of chimeric nucleotide sequences. **(A)** Overview of nucleotide sequences for CaCYP716E11_UnOP, Chimera1, and Chimera2. **(B)** Arjunolic acid production by expression of CaCYP716E11_UnOP, Chimera1, or Chimera2 in the maslinic acid-producing strain HiMas. **(C)** Overview of nucleotide sequences for CaCYP716E11_ExOP, Chimera3, and Chimera4. **(D)** Arjunolic acid production by expression of CaCYP716E11_UnOP, Chimera3, or Chimera4 in the maslinic acid-producing strain HiMas. Averages and standard deviations are based on triplicate cultivations 24-deep well plates with YPD80-media. Asterisks indicate statistical significant differences (*t*-test, critical two-tailed, p < 0.05). N, N-terminus. TM, transmembrane domain.

Swapping of cytochrome P450 N-terminal segments has been shown to sometimes increase expression in *S. cerevisiae* (Cabello-Hurtado et al., 1999). Codon-optimization of only the 5′-end of cytochrome P450 genes has also increased expression in *S. cerevisiae* (Batard et al., 2000). Therefore, we attempted to replace the NTM-domain of *KsCYP72A397_UnOP* and *KsCYP72A397_ExOP* with the one from *CaCYP714E19_IhOP* or *KsCYP72A397_IhOP* (Fig. S8). The domains were predicted with InterProScan (Jones et al., 2014). Yet, none of the NTM-swapped *KsCYP72A397* variants resulted in arjunolic acid production when expressed in HiMas. Conversely, we investigated whether swapping the NTM-domain from *CaCYP714E19_UnOP* and *CaCYP714E19_ExOP* with the NTM-domain either from *CaCYP714E19_IhOP* or *KsCYP72A397_IhOP* would affect arjunolic acid production. Replacing the NTM-domain of *CaCYP714E19_UnOP* with either the NTM-domain from *CaCYP714E19_IhOP* (*Chimera1*) or *KsCYP72A397_IhOP* (*Chimera2*) seemingly improved arjunolic acid titers compared to *CaCYP714E19_UnOP* when expressed in HiMas (Fig. 5). More strikingly, replacing the NTM-domain of *CaCYP714E19_ExOP* with either the NTM-domain from *CaCYP714E19_IhOP* (*Chimera3*) or *KsCYP72A397_IhOP* (*Chimera4*) gave rise to 105.2 ± 16.0 mg/L and 4.0 ± 0.16 mg/g DCW, or 182.0 ± 19.0 mg/L and 9.1 ± 0.3 mg/g DCW arjunolic acid, respectively. While expression of Chimera3 in HiMas produced arjunolic acid similar to the expression of *CaCYP714E19_IhOP* (biomass specific yield, *p* > 0.05), expression of *Chimera4* in HiMas produced significantly more arjunolic acid than both the *Chimera3* and *CaCYP714E19_IhOP* expressing strains (biomass specific yield, *p* < 0.05). Small amounts of maslinic acid were detected for the *Chimera4*-expressing strain, but not for the strains expressing *CaCYP714E19_ExOP* or *Chimera3*, which suggested that Chimera4p had a ‘pulling’ effect on the triterpenoid pathway when expressed in HiMas (Fig. S9).

## 4 Discussion

Previous studies have demonstrated the high production of ursane and oleanane triterpenoids in yeast. For example, 1107.9 mg/L α-amyrin, or 384 mg/L maslinic acid was achieved in engineered *S. cerevisiae*, and 540.7 mg/L oleanolic acid in engineered *Y. lipolytica* (Dai et al., 2014, 2019; Li et al., 2020; Yu et al., 2020). Like the C_30_-platform strain used in this study, the above studies featured overexpression of *SQS*, and *HMG* versions, while *ERG20* overexpression was used for oleanolic acid and α-amyrin production, and *SQE* overexpression was used for α-amyrin and maslinic acid production. This suggests that alleviating rate-limiting MVA-pathway steps and increasing flux towards 2,3-oxidosqualene are potent strategies for developing yeast triterpenoid cell factories. It was also demonstrated that expression of *Cucurbita pepo* SQE in *S. cerevisiae* and *Nicotiana benthamiana* could improve the production of 2,3-oxidosqualene and thereof-derived triterpenoids (Dong et al., 2018). Furthermore, our previous study showed that although the C_30_-platform strain produced improved amounts of 2,3-oxidosqualene, it still accumulated 262.7 mg/L squalene (Arnesen et al., 2020). Therefore, increasing the copy numbers of SQE was an obvious strategy to improve triterpenoid production in the C_30_-platform strain since the beforementioned studies also utilized multi-expression of triterpenoid and MVA-biosynthetic genes (Dai et al., 2014, 2019; Li et al., 2020; Yu et al., 2020).

Several studies have investigated squalene production in engineered *Y. lipolytica* strains, demonstrating titers in the range of 205–531.6 mg/L (Arnesen et al., 2020; Gao et al., 2017; H. Liu et al., 2020; Wei et al., 2021). Interestingly, deletion of the peroxisomal biogenesis factor 10 gene (*ΔPEX10*) or overexpression of diacylglycerol acyltransferase gene (*DGA1*) both increased squalene and lipid accumulation considerably. Manipulating fatty acid synthesis and degradation may also be useful for triterpenoid production. Notably, an *S. cerevisiae* squalene overproducing strain was constructed by targeting the MVA- and squalene biosynthesis pathways to the peroxisomes and improving the peroxisomal ATP, NADPH and acetyl-CoA pools, resulting in 1312.8 mg/L and 284.5 mg/g DCW squalene (G. S. Liu et al., 2020). Hybridization of the peroxisomally engineered strain with a cytoplasmically engineered strain, and fed-batch fermentation of the resulting strain provided 11 g/L squalene. Compartmentalization engineering could also prove advantageous for complex triterpenoid production in *Y. lipolytica*.

Fusing proteins can improve their expression, folding, and enzymatic activity (Guo et al., 2017). Yet, the fusion of MdOSC^m^p with trYlSQE^Δ1-37^p did not benefit ursolic acid production, but fusing GgBASp and trYlSQE^Δ1-37^p greatly improved oleanolic acid production, which showed that fusion of similar enzymes can lead to different outcomes. SQEp is a flavin adenine dinucleotide (FAD) containing monooxygenase, and it localizes to the ER and lipid droplets in *S. cerevisiae* (Leber et al., 1998). Little is known about the exact subcellular location of GgBASp, but the locations of other oxidosqualene cyclases have been investigated. The *Panax ginseng* dammarenediol synthase and *A. thaliana* cycloartenol synthase associate mainly with lipid particles and, to a lesser extent, the ER when expressed in *S. cerevisiae*, despite their lack of transmembrane domains (Liang et al., 2012; Milla et al., 2003). The *A. thaliana* marneral synthase was shown to localize to the ER (Go et al., 2012). One study indicated physical interaction between *Cucurbita pepo* cucurbitadienol synthase and the ER-localized *C. pepo* squalene epoxidase 2 *in planta* (Dong et al., 2018). Likewise, the *Ononis spinosa* α-onocerin synthase was shown to solubilize in the cytoplasm and interact with the ER-localized *O. spinosa* squalene epoxidases 1 and 2 *in planta* (Almeida et al., 2018). These studies suggest that fusing triterpenoid synthases and SQEps could mimic natural interactions. It would be interesting to investigate how the protein fusion strategies from this study influence the enzymes' subcellular location, expression, and catalytic rate. The length of the linker can also affect the activity of the fusion protein. For example, the short GSG linker was found to be most effective for fusing *A. thaliana* 4-coumaroyl-CoA ligase and *Vitis vinifera* stilbene synthase, while using four GSG linkers was best for linking *Streptomyces clavuligerus* 1,8-cineole synthase and the *Citrobacter braakii* CYP176A1 (Guo et al., 2017; Wang et al., 2021). Therefore, optimization of linker length could potentially improve the activity of the fusion proteins described in this study.

Expression of *C. asiatica* cytochromes P450 in the ursolic acid-producing strain led to asiatic and madecassic acid production. To our knowledge, this is the first report of heterologously produced asiatic and madecassic acids. However, since only 3.1 ± 0.03 mg/L madecassic acid was produced, further optimization is needed before heterologous production becomes an industrially viable option.

We also applied the same and similar cytochromes P450 to produce oleananes, initially developing oleanolic and maslinic acids producing strains. However, pathway extension by expression of any *KsCYP72A397*-codon variant in HiMas did not lead to the formation of arjunolic acid. *KsCYP72A397* PCR-amplified from cDNA had previously been expressed *S. cerevisiae*, which led to the C-23 hydroxylation of oleanolic acid, which produced hederagenin (Han et al., 2018). The *S. cerevisiae* strain was based on the WAT21 strain, which expressed the cytochrome P450 reductase AtATR2p (Urban et al., 1997). Our *Y. lipolytica* strains also expressed AtATR2p, so the lack of KsCYP72A397p activity is probably not due to insufficient reductase partnering. Furthermore, it may be that some cytochromes P450 seemingly work in *S. cerevisiae* but not in *Y. lipolytica*; In one report, *Vitis vinifera* CYP716A17p could catalyze the C-28 carboxylation of β-amyrin into oleanolic acid when expressed in *S. cerevisiae* microsomes (Fukushima et al., 2011). However, the expression of VvCYP716A17p in *Y. lipolytica* did not lead to the C-28 carboxylation of lupeol into betulinic acid (Jin et al., 2019). Therefore, the lack of KsCYP72A397p activity could be due to inherent differences between *Y. lipolytica* and *S. cerevisiae*.

We improved the production of arjunolic acid by codon optimizing *CaCYP714E19* with an algorithm that heavily favored highly expressed *Y. lipolytica* codons, which outperformed an algorithm from an external source. Other studies have also found that different codon-optimization algorithms lead to variable outcomes in protein expression (Ranaghan et al., 2021). Furthermore, we demonstrate that optimizing the 5’ end of *CaCYP714E19* with highly favored codons can lead to improved arjunolic acid production. N-terminal codon optimization has previously been used to increase the expression of plant cytochromes P450 in yeast. For example, optimization of the first 18, 39, or 111 nucleotides of the wheat *CYP73A17*-DNA sequence led to an expression increase in *S. cerevisiae* proportional to the optimized nucleotide segment length. At the same time, the non-optimized version did not seem to express at all (Batard et al., 2000). Likewise, optimization of the 120 first nucleotides of wheat *CYP86A5* also led to increased expression in *S. cerevisiae*.

Microsomal eukaryotic cytochromes P450 are typically anchored in the ER by an NTM-domain, while the heme catalytic site occurs further towards the C-terminal (Poulos and Johnson, 2015). While the sequence and architecture of the heme catalytic site are important for enzyme function, several studies have demonstrated that some cytochromes P450 retain catalytic activity upon N-terminal truncation (Cosme and Johnson, 2000; Cullin, 1992; Von Wachenfeldt et al., 1997). Therefore, NTM-domain engineering could increase cytochrome P450 expression and activity without altering its basic function. Indeed, N-terminal modifications, often featuring truncations, tagging, or substitutions, of eukaryotic cytochromes P450 are common strategies for improving expression in prokaryotic systems (Christensen et al., 2017; Vazquez-Albacete et al., 2017). However, examples of N-terminal modifications improving cytochrome P450 expression in yeast are scarce. One report showed that when the N-terminal of wheat CYP51 was swapped with either the N-terminal from sorghum or *S. cerevisiae* CYP51 and both were expressed in *S. cerevisiae*; the sorghum-wheat CYP51 chimera had improved expression compared to yeast-wheat CYP51 chimera (Cabello-Hurtado et al., 1999). Another report demonstrated increased artemisinic aldehyde production in yeast when the first 15 amino acid residues from the *Artemisia annua* cytochrome P450 and reductase fusion sequence (AaCYP71AV1-CPRp) were replaced with a short N-terminal hydrophilic bovine protein tag (Chen et al., 2017). Likewise, our results show that exchanging the NTM-domain of *CaCYP714E19_ExOP* with *KsCYP72A397_IhOP* led to highly improved arjunolic acid production. This result suggests that N-terminal modification of eukaryotic cytochromes P450 is a potential strategy for increasing the production of small oxygenated molecules in yeast. 182.0 ± 19.0 mg/L arjunolic acid was produced when the *Chimera4*-sequence was expressed in HiMas. Arjunolic acid can be extracted from the bark of *Terminalia arjuna* with yields ranging from 0.1 to 0.524% w/w (Kalola and Rajani, 2006; Ramesh et al., 2012). Simple ethanolic extraction of our final arjunolic acid-producing strain yielded 9.1 ± 0.3 mg/g DCW (0.91% w/dw), which suggests that engineering *Y. lipolytica* cell factories may be a promising way to produce arjunolic acid in the future.

In summary, we report the first-time heterologous production of the triterpenoids asiatic, madecassic, and arjunolic acid. Further improvement of the yeast chassis and fermentation conditions can result in economically viable and sustainable industrial processes for producing complex oleanane and ursane triterpenoids.

## Author statements

Jonathan Asmund Arnesen: Conceptualization, methodology, investigation, validation, formal analysis, visualization, writing – original draft, writing – review & editing, supervision. Arian Belmonte Del Ama: Methodology, investigation, validation, formal analysis, writing – review & editing. Sidharth Jayachandran: Methodology, investigation, validation, formal analysis, writing – review & editing. Jonathan Dahlin: Methodology, software, writing – review & editing. Daniela Rago: Methodology, investigation, formal analysis, visualization, writing – review & editing. Aaron John Christian Andersen: Methodology, investigation, formal analysis, writing – review & editing. Irina Borodina: Conceptualization, methodology, supervision, writing – original draft, writing – review & editing, funding acquisition.

## Funding

The research was funded by the 10.13039/501100009708Novo Nordisk Foundation (grant agreements NNF15OC0016592, NNF20CC0035580, and NNF20OC0060809) and by the European Union's Horizon 2020 research and innovation programme under grant agreement No. 760798 (OLEFINE).

## Declaration of competing interest

The authors declare that they have no known competing financial interests or personal relationships that could have appeared to influence the work reported in this paper.

## References

[bib1] Almeida A., Dong L., Khakimov B., Bassard J.-E., Moses T., Lota F., Goossens A., Appendino G., Bak S. (2018). A single oxidosqualene cyclase produces the *seco*-triterpenoid α-onocerin. Plant Physiol..

[bib2] Andre C.M., Legay S., Deleruelle A., Nieuwenhuizen N., Punter M., Brendolise C., Cooney J.M., Lateur M., Hausman J.F., Larondelle Y., Laing W.A. (2016). Multifunctional oxidosqualene cyclases and cytochrome P450 involved in the biosynthesis of apple fruit triterpenic acids. New Phytol..

[bib3] Arnesen J.A., Kildegaard K.R., Cernuda Pastor M., Jayachandran S., Kristensen M., Borodina I. (2020). *Yarrowia lipolytica* strains engineered for the production of terpenoids. Front. Bioeng. Biotechnol..

[bib4] Batard Y., Hehn A., Nedelkina S., Schalk M., Pallett K., Schaller H., Werck-Reichhart D. (2000). Increasing expression of P450 and P450-reductase proteins from monocots in heterologous systems. Arch. Biochem. Biophys..

[bib5] Beopoulos A., Cescut J., Haddouche R., Uribelarrea J.L., Molina-Jouve C., Nicaud J.M. (2009). *Yarrowia lipolytica* as a model for bio-oil production. Prog. Lipid Res..

[bib6] Bishayee A., Ahmed S., Brankov N., Perloff M. (2011). Triterpenoids as potential agents for the chemoprevention and therapy of breast cancer. Front. Biosci..

[bib7] Bylka W., Znajdek-Awiżeń P., Studzińska-Sroka E., Brzezińska M. (2013). *Centella asiatica* in cosmetology. Adv. Dermatol. Allergol..

[bib8] Cabello-Hurtado F., Taton M., Forthoffer N., Kahn R., Søren B., Rahier A., Werck-Reichhart D. (1999). Optimized expression and catalytic properties of a wheat obtusifoliol 14α-demethylase (CYP51) expressed in yeast complementation of *erg11Δ* yeast mutants by plant CYP51. Eur. J. Biochem..

[bib9] Carelli M., Biazzi E., Panara F., Tava A., Scaramelli L., Porceddu A., Graham N., Odoardi M., Piano E., Arcioni S., May S., Scotti C., Calderini O. (2011). *Medicago truncatula* CYP716A12 is a multifunctional oxidase involved in the biosynthesis of hemolytic saponins. Plant Cell.

[bib10] Chen X., Zhang C., Too H.P. (2017). Multienzyme biosynthesis of dihydroartemisinic acid. Molecules.

[bib11] Christen S., Sauer U. (2011). Intracellular characterization of aerobic glucose metabolism in seven yeast species by ^13^C flux analysis and metabolomics. FEMS Yeast Res..

[bib12] Christensen U., Vazquez-Albacete D., Søgaard K.M., Hobel T., Nielsen M.T., Harrison S.J., Hansen A.H., Møller B.L., Seppälä S., Nørholm M.H.H. (2017). De-bugging and maximizing plant cytochrome P450 production in *Escherichia coli* with C-terminal GFP fusions. Appl. Microbiol. Biotechnol..

[bib13] Cosme J., Johnson E.F. (2000). Engineering microsomal cytochrome P450 2C5 to be a soluble, monomeric enzyme: mutations that alter aggregation, phospholipid dependence of catalysis, and membrane binding. J. Biol. Chem..

[bib14] Cullin C. (1992). Two distinct sequences control the targeting and anchoring of the mouse P450 1A1 into the yeast endoplasmic reticulum membrane. Biochem. Biophys. Res. Commun..

[bib15] Dahlin J., Holkenbrink C., Marella E.R., Wang G., Liebal U., Lieven C., Weber D., McCloskey D., Wang H.-L., Ebert B.E., Herrgård M.J., Blank L.M., Borodina I. (2019). Multi-omics analysis of fatty alcohol production in engineered yeasts *Saccharomyces cerevisiae* and *Yarrowia lipolytica*. Front. Genet..

[bib16] Dai Z., Liu Y., Sun Z., Wang D., Qu G., Ma X., Fan F., Zhang L., Li S., Zhang X. (2019). Identification of a novel cytochrome P450 enzyme that catalyzes the C-2α hydroxylation of pentacyclic triterpenoids and its application in yeast cell factories. Metab. Eng..

[bib17] Dai Z., Wang B., Liu Y., Shi M., Wang D., Zhang Xianan, Liu T., Huang L., Zhang Xueli (2014). Producing aglycons of ginsenosides in bakers' yeast. Sci. Rep..

[bib18] Dong L., Pollier J., Bassard J.E., Ntallas G., Almeida A., Lazaridi E., Khakimov B., Arendt P., de Oliveira L.S., Lota F., Goossens A., Michoux F., Bak S. (2018). Co-expression of squalene epoxidases with triterpene cyclases boosts production of triterpenoids in plants and yeast. Metab. Eng..

[bib19] Dujon B., Sherman D., Fischer G., Durrens P., Casaregola S., Lafontaine I., de Montigny J., Marck C., Neuvéglise C., Talla E., Goffard N., Frangeul L., Aigle M., Anthouard V., Babour A., Barbe V., Barnay S., Blanchin S., Beckerich J., Beyne E., Bleykasten C., Boisramé A., Boyer J., Cattolico L., Confanioleri F., de Daruvar A., Despons L., Fabre E., Fairhead C., Ferry-Dumazet H., Groppi A., Hantraye F., Hennequin C., Jauniaux N., Joyet P., Kachouri R., Kerrest A., Koszul R., Lemaire M., Lesur I., Ma L., Muller H., Nicaud J.-M., Nikolski M., Oztas S., Ozier-Kalogeropoulos O., Pellenz S., Potier S., Richard G.-F., Straub M.-L., Suleau A., Swennen D., Tekaia F., Wésolowski-Louvel M., Westhof E., Wirth B., Zeniou-Meyer M., Zivanovic I., Bolotin-Fukuhara M., Thierry A., Bouchier C., Caudron B., Scarpelli C., Gaillardin C., Weissenbach J., Wincker P., Souciet J.-L. (2004). Genome evolution in yeasts. Nature.

[bib20] Englund E., Andersen-Ranberg J., Miao R., Hamberger B., Lindberg P. (2015). Metabolic engineering of *synechocystis* sp. PCC 6803 for production of the plant diterpenoid manoyl oxide. ACS Synth. Biol..

[bib21] Fath S., Bauer A.P., Liss M., Spriestersbach A., Maertens B., Hahn P., Ludwig C., Schäfer F., Graf M., Wagner R. (2011). Multiparameter RNA and codon optimization: a standardized tool to assess and enhance autologous mammalian gene expression. PLoS One.

[bib22] Fukushima E.O., Seki H., Ohyama K., Ono E., Umemoto N., Mizutani M., Saito K., Muranaka T. (2011). CYP716A subfamily members are multifunctional oxidases in triterpenoid biosynthesis. Plant Cell Physiol..

[bib23] Fukushima E.O., Seki H., Sawai S., Suzuki M., Ohyama K., Saito K., Muranaka T. (2013). Combinatorial biosynthesis of legume natural and rare triterpenoids in engineered yeast. Plant Cell Physiol..

[bib24] Gao S., Tong Y., Zhu L., Ge M., Zhang Y., Chen D., Jiang Y., Yang S. (2017). Iterative integration of multiple-copy pathway genes in *Yarrowia lipolytica* for heterologous β-carotene production. Metab. Eng..

[bib25] Ghosh J., Sil P.C. (2013). Arjunolic acid: a new multifunctional therapeutic promise of alternative medicine. Biochimie.

[bib26] Go Y.S., Lee S.B., Kim H.J., Kim J., Park H.Y., Kim J.K., Shibata K., Yokota T., Ohyama K., Muranaka T., Arseniyadis S., Suh M.C. (2012). Identification of marneral synthase, which is critical for growth and development in *Arabidopsis*. Plant J..

[bib27] Gomes J., Pereira T., Vilarinho C., Duarte M.D.L., Brito C. (2010). Contact dermatitis due to *Centella asiatica*. Contact Dermatitis.

[bib28] González-Coloma A., López-Balboa C., Santana O., Reina M., Fraga B.M. (2011). Triterpene-based plant defenses. Phytochemistry Rev..

[bib29] Guo H., Yang Y., Xue F., Zhang H., Huang T., Liu W., Liu H., Zhang F., Yang M., Liu C., Lu H., Zhang Y., Ma L. (2017). Effect of flexible linker length on the activity of fusion protein 4-coumaroyl-CoA ligase::stilbene synthase. Mol. Biosyst..

[bib30] Han J.Y., Chun J.H., Oh S.A., Park S.B., Hwang H.S., Lee H., Choi Y.E. (2018). Transcriptomic analysis of *Kalopanax septemlobus* and characterization of KsBAS, CYP716A94 and CYP72A397 genes involved in hederagenin saponin biosynthesis. Plant Cell Physiol..

[bib31] Hayashi H., Huang P., Kirakosyan A., Inoue K., Hiraoka N., Ikeshiro Y., Kushiro T., Shibuya M., Ebizuka Y. (2001). Cloning and characterization of a cDNA encoding β-amyrin synthase involved in glycyrrhizin and soyasaponin biosyntheses in licorice. Biol. Pharm. Bull..

[bib32] Hemalatha T., Pulavendran S., Balachandran C., Manohar B.M., Puvanakrishnan R. (2010). Arjunolic acid: a novel phytomedicine with multifunctional therapeutic applications. Indian J. Exp. Biol..

[bib33] Holkenbrink C., Dam M.I., Kildegaard K.R., Beder J., Dahlin J., Doménech Belda D., Borodina I. (2018). EasyCloneYALI: CRISPR/Cas9-Based synthetic toolbox for engineering of the yeast *Yarrowia lipolytica*. Biotechnol. J..

[bib34] Hu T., Zhou J., Tong Y., Su P., Li X., Liu Y., Liu N., Wu X., Zhang Y., Wang J., Gao L., Tu L., Lu Y., Jiang Z., Zhou Y.J., Gao W., Huang L. (2020). Engineering chimeric diterpene synthases and isoprenoid biosynthetic pathways enables high-level production of miltiradiene in yeast. Metab. Eng..

[bib35] Idris F.N., Mohd Nadzir M. (2021). Comparative studies on different extraction methods of *Centella asiatica* and extracts bioactive compounds effects on antimicrobial activities. Antibiotics.

[bib36] Jin C.-C., Zhang J.-L., Song H., Cao Y.-X. (2019). Boosting the biosynthesis of betulinic acid and related triterpenoids in *Yarrowia lipolytica* via multimodular metabolic engineering. Microb. Cell Factories.

[bib37] Jones P., Binns D., Chang H.-Y., Fraser M., Li W., McAnulla C., McWilliam H., Maslen J., Mitchell A., Nuka G., Pesseat S., Quinn A.F., Sangrador-Vegas A., Scheremetjew M., Yong S.-Y., Lopez R., Hunter S. (2014). InterProScan 5: genome-scale protein function classification. Bioinformatics.

[bib38] Jorge O.A., Jorge A.D. (2005). Hepatotoxicity associated with the ingestion of *Centella asiatica*. Rev. Esp. Enferm. Dig..

[bib39] Kalola J., Rajani M. (2006). Extraction and TLC desitometric determination of triterpenoid acids (arjungenin, arjunolic acid) from *Terminalia arjuna* stem bark without interference of Tannins. Chromatographia.

[bib40] Kildegaard K.R., Adiego-Pérez B., Doménech Belda D., Khangura J.K., Holkenbrink C., Borodina I. (2017). Engineering of *Yarrowia lipolytica* for production of astaxanthin. Synth. Syst. Biotechnol..

[bib41] Kildegaard K.R., Arnesen J.A., Adiego-Pérez B., Rago D., Kristensen M., Klitgaard A.K., Hansen E.H., Hansen J., Borodina I. (2021). Tailored biosynthesis of gibberellin plant hormones in yeast. Metab. Eng..

[bib42] Kim O.T., Um Y., Jin M.L., Kim J.U., Hegebarth D., Busta L., Racovita R.C., Jetter R. (2018). A novel multifunctional C-23 Oxidase, CYP714E19, is involved in asiaticoside biosynthesis. Plant Cell Physiol..

[bib43] Leber R., Landl K., Zinser E., Ahorn H., Spök A., Kohlwein S.D., Turnowsky F., Daum G. (1998). Dual localization of squalene epoxidase, Erg1p, in yeast reflects a relationship between the endoplasmic reticulum and lipid particles. Mol. Biol. Cell.

[bib44] Li D., Wu Y., Wei P., Gao X., Li M., Zhang C., Zhou Z., Lu W. (2020). Metabolic engineering of *Yarrowia lipolytica* for heterologous oleanolic acid production. Chem. Eng. Sci..

[bib45] Liang Y.L., Zhao S.J., Xu L.X., Zhang X.Y. (2012). Heterologous expression of dammarenediol synthase gene in an engineered *Saccharomyces cerevisiae*. Lett. Appl. Microbiol..

[bib46] Liao P., Hemmerlin A., Bach T.J., Chye M. (2016). The potential of the mevalonate pathway for enhanced isoprenoid production. Biotechnol. Adv..

[bib47] Liu G.S., Li T., Zhou W., Jiang M., Tao X.Y., Liu M., Zhao M., Ren Y.H., Gao B., Wang F.Q., Wei D.Z. (2020). The yeast peroxisome: a dynamic storage depot and subcellular factory for squalene overproduction. Metab. Eng..

[bib48] Liu H., Wang F., Deng L., Xu P. (2020). Genetic and bioprocess engineering to improve squalene production in *Yarrowia lipolytica*. Bioresour. Technol..

[bib49] Madeira F., Park Y.M., Lee J., Buso N., Gur T., Madhusoodanan N., Basutkar P., Tivey A.R.N., Potter S.C., Finn R.D., Lopez R. (2019). The EMBL-EBI search and sequence analysis tools APIs in 2019. Nucleic Acids Res..

[bib50] Marella E.R., Dahlin J., Dam M.I., ter Horst J., Christensen H.B., Sudarsan S., Wang G., Holkenbrink C., Borodina I. (2020). A single-host fermentation process for the production of flavor lactones from non-hydroxylated fatty acids. Metab. Eng..

[bib51] Miettinen K., Pollier J., Buyst D., Arendt P., Csuk R., Sommerwerk S., Moses T., Mertens J., Sonawane P.D., Pauwels L., Aharoni A., Martins J., Nelson D.R., Goossens A. (2017). The ancient CYP716 family is a major contributor to the diversification of eudicot triterpenoid biosynthesis. Nat. Commun..

[bib52] Milla P., Viola F., Oliaro-Bosso S., Rocco F., Cattel L., Joubert B.M., LeClair R.J., Matsuda S.P.T., Ballianoa G. (2003). Subcellular localization of oxidosqualene cyclases from *Arabidopsis thaliana, Trypanosoma cruzi*, and *Pneumocystis carinii* expressed in yeast. Lipids.

[bib53] Mora-Pale M., Sanchez-Rodriguez S.P., Linhardt R.J., Dordick J.S., Koffas M.A. (2014). Biochemical strategies for enhancing the *in vivo* production of natural products with pharmaceutical potential. Curr. Opin. Biotechnol..

[bib54] Poulos T.L., Johnson E.F. (2015). Cytochrome P450.

[bib55] Ramesh A.S., Christopher J.G., Radhika R., Setty C.R., Thankamani V. (2012). Isolation, characterisation and cytotoxicity study of arjunolic acid from *Terminalia arjuna*. Nat. Prod. Res..

[bib56] Ranaghan M.J., Li J.J., Laprise D.M., Garvie C.W. (2021). Assessing optimal: inequalities in codon optimization algorithms. BMC Biol..

[bib57] Ríos J.-L. (2010). Effects of triterpenes on the immune system. J. Ethnopharmacol..

[bib58] Sun B., Wu L., Wu Y., Zhang C., Qin L., Hayashi M., Kudo M., Gao M., Liu T. (2020). Therapeutic potential of *Centella asiatica* and its triterpenes: a review. Front. Pharmacol..

[bib59] Suzuki H., Fukushima E.O., Umemoto N., Ohyama K., Seki H., Muranaka T. (2018). Comparative analysis of CYP716A subfamily enzymes for the heterologous production of C-28 oxidized triterpenoids in transgenic yeast. Plant Biotechnol..

[bib60] Swainston N., Currin A., Day P.J., Kell D.B. (2014). GeneGenie: optimized oligomer design for directed evolution. Nucleic Acids Res..

[bib61] Turck D., Castenmiller J., de Henauw S., Hirsch‐Ernst K., Kearney J., Maciuk A., Mangelsdorf I., McArdle H.J., Naska A., Pelaez C., Pentieva K., Siani A., Thies F., Tsabouri S., Vinceti M., Cubadda F., Engel K., Frenzel T., Heinonen M., Marchelli R., Neuhäuser‐Berthold M., Pöting A., Poulsen M., Sanz Y., Schlatter J.R., van Loveren H., Ackerl R., Knutsen H.K. (2019). Safety of *Yarrowia lipolytica* yeast biomass as a novel food pursuant to Regulation (EU) 2015/2283. EFSA J..

[bib62] Untergasser A., Cutcutache I., Koressaar T., Ye J., Faircloth B.C., Remm M., Rozen S.G. (2012). Primer3—new capabilities and interfaces. Nucleic Acids Res..

[bib63] Urban P., Mignotte C., Kazmaier M., Delorme F., Pompon D. (1997). Cloning, yeast expression, and characterization of the coupling of two distantly related *Arabidopsis thaliana* NADPH-cytochrome P450 reductases with P450 CYP73A5. J. Biol. Chem..

[bib64] Vazquez-Albacete D., Cavaleiro A.M., Christensen U., Seppälä S., Møller B.L., Nørholm M.H.H. (2017). An expression tag toolbox for microbial production of membrane bound plant cytochromes P450. Biotechnol. Bioeng..

[bib65] Von Wachenfeldt C., Richardson T.H., Cosme J., Johnson E.F. (1997). Microsomal P450 2C3 is expressed as a soluble dimer in *Escherichia coli* following modifications of its N-terminus. Arch. Biochem. Biophys..

[bib66] Wang C.M., Chen H.T., Li T.C., Weng J.H., Jhan Y.L., Lin S.X., Chou C.H. (2014). The role of pentacyclic triterpenoids in the allelopathic effects of *Alstonia scholaris*. J. Chem. Ecol..

[bib67] Wang X., Pereira J.H., Tsutakawa S., Fang X., Adams P.D., Mukhopadhyay A., Lee T.S. (2021). Efficient production of oxidized terpenoids via engineering fusion proteins of terpene synthase and cytochrome P450. Metab. Eng..

[bib68] Wei L.J., Cao X., Liu J.J., Kwak S., Jin Y.S., Wang W., Hua Q. (2021). Increased accumulation of squalene in engineered *Yarrowia lipolytica* through deletion of *PEX10* and *URE2*. Appl. Environ. Microbiol..

[bib69] Yu Y., Rasool A., Liu H., Lv B., Chang P., Song H., Wang Y., Li C. (2020). Engineering *Saccharomyces cerevisiae* for high yield production of α-amyrin via synergistic remodeling of α-amyrin synthase and expanding the storage pool. Metab. Eng..

