# Supplementary material for: Engineering of Yarrowia lipolytica for the production of plant triterpenoids: Asiatic, madecassic, and arjunolic acids
Source: Metab Eng Commun. 2022 Mar 26;14:e00197. doi: 10.1016/j.mec.2022.e00197 (PMC9011116; doi:10.1016/j.mec.2022.e00197)
Supplement: Multimedia component 1 [file mmc1.docx]

## Supplementary figures


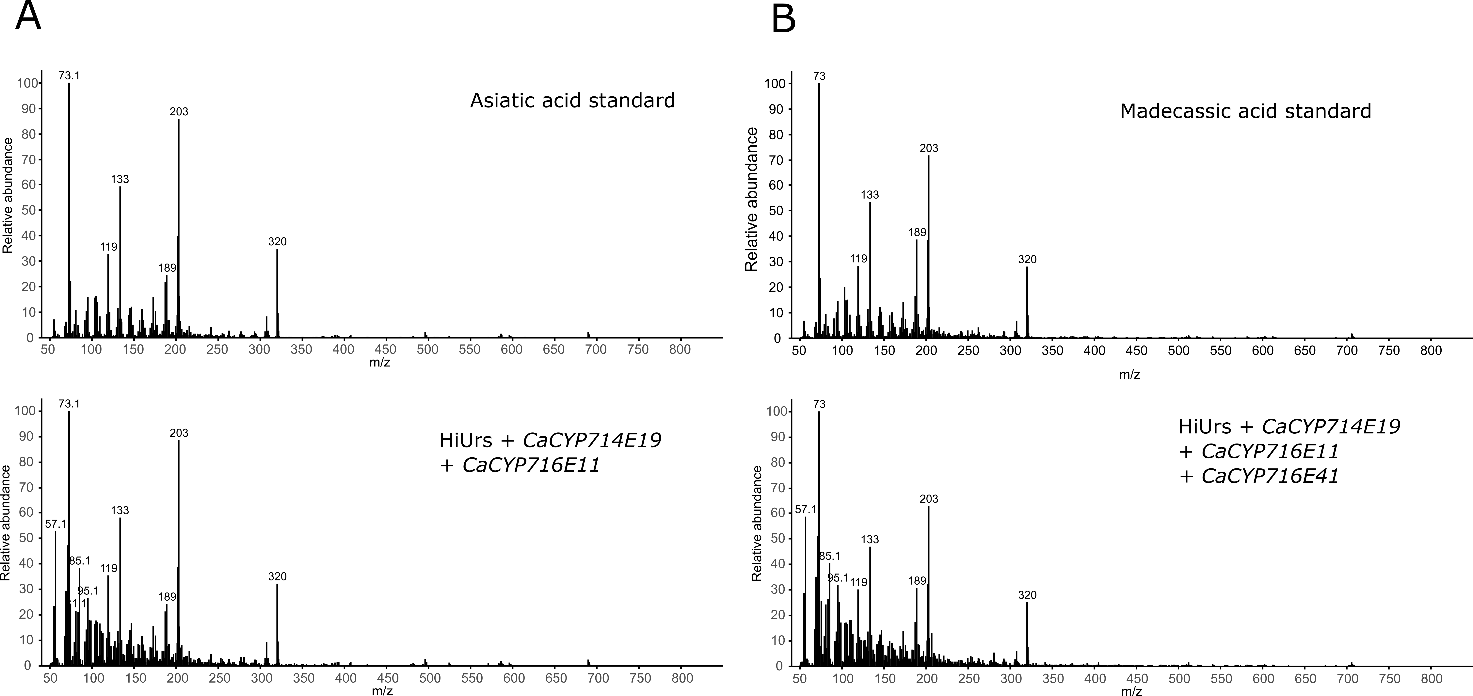


Supplementary Figure 1: GC/MS Spectra of triterpenoid peaks from derivatized yeast samples and authentic standards. **(A)** Derivatized asiatic acid standard peak with retention time 23.3 min (top) and derivatized yeast sample with retention time (RT) 23.3 min (bottom). **(B)** Derivatized madecassic acid standard peak with RT: 27.4 min (top) and derivatized yeast sample with RT: 27.5 min (bottom).


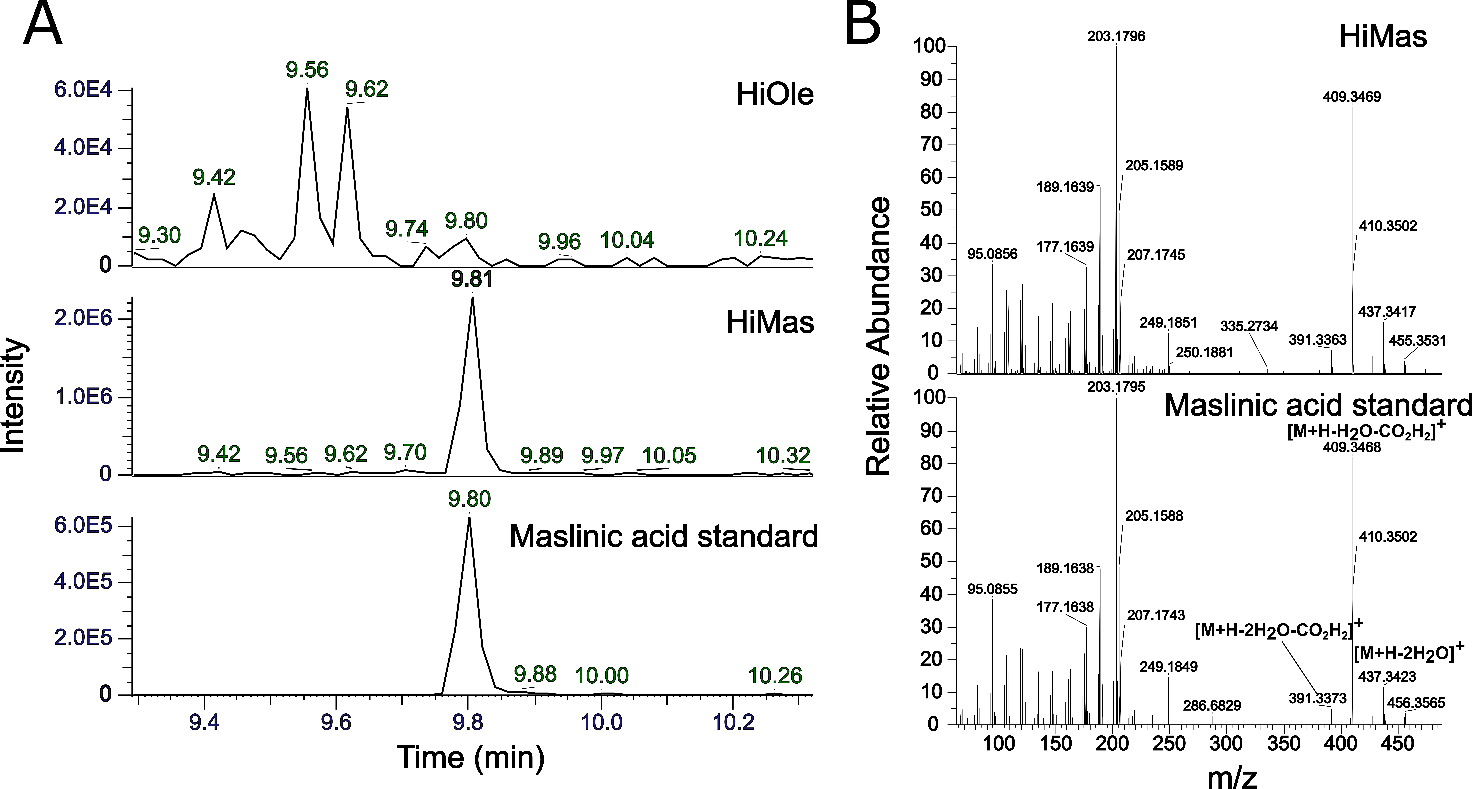


Figure 2: LC-MS analysis for the presence of maslinic acid. **(A)**Extracted ion chromatograms (EICs) of control (HiOle), HiMas, and an authentic maslinic acid standard. **(B)** comparison of MS2 spectra from HiMas at RT: 9.81 min and the authentic standard RT: 9.81 min.


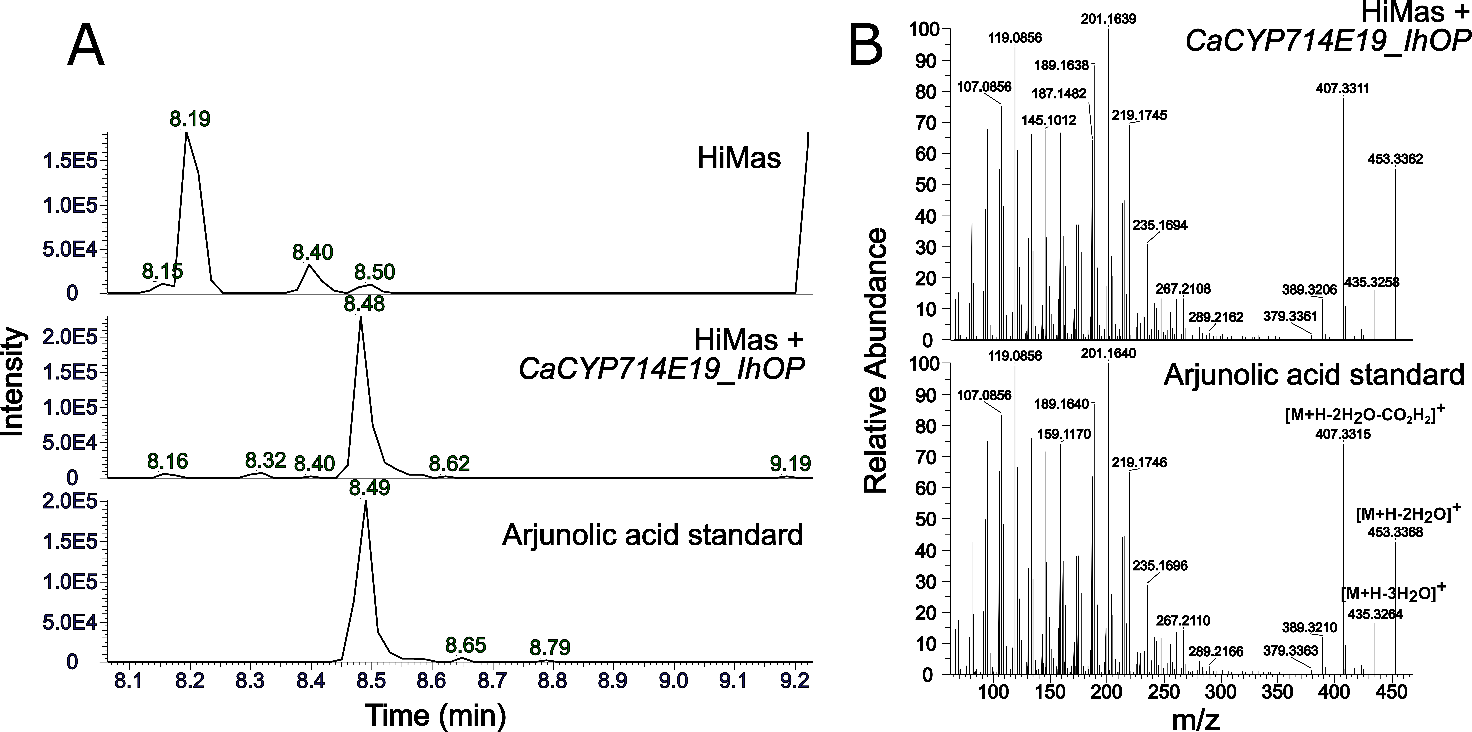


Figure 3: LC-MS analysis for the presence of arjunolic acid. **(A)** Extracted ion chromatograms of control (HiMas), HiMas expressing CaCYP714E19_IhOP, and an authentic arjunolic acid standard. **(B)** comparison of MS2 spectra from HiMas expressing CaCYP714E19_IhOP at RT: 8.48 min and the authentic standard at RT: 8.48 min..


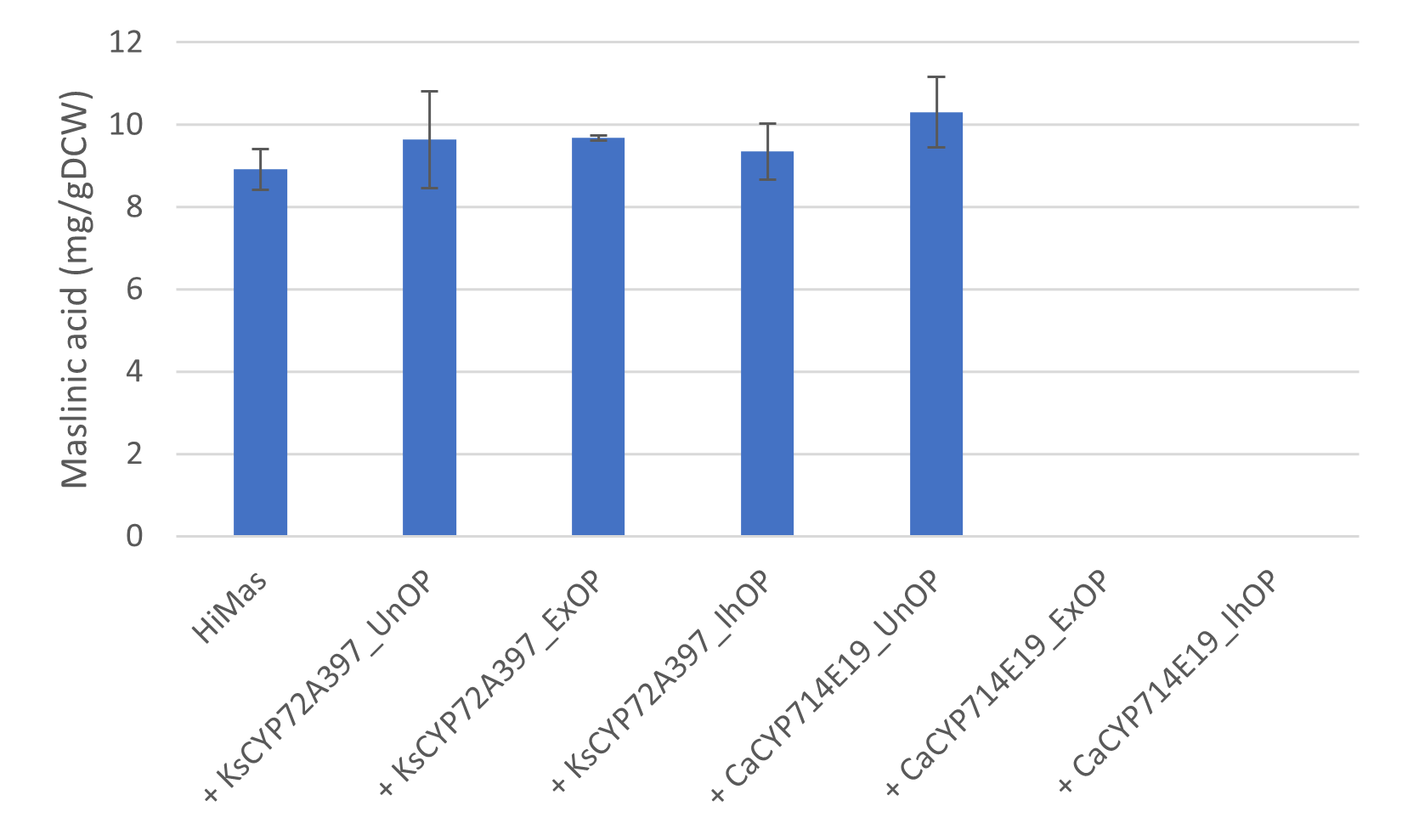


Supplementary Figure 4: Maslinic acid production by HiMas-based strains expressing KsCYP72A397 or CaCYP714E19. UnOP, not codon optimized for Y. lipolytica. ExOp, codon optimized for Y. lipolytica using an external algorithm. IhOp, codon optimized for Y. lipolytica using an in-house algorithm. All titer averages and standard deviations are based three experimental replicates.


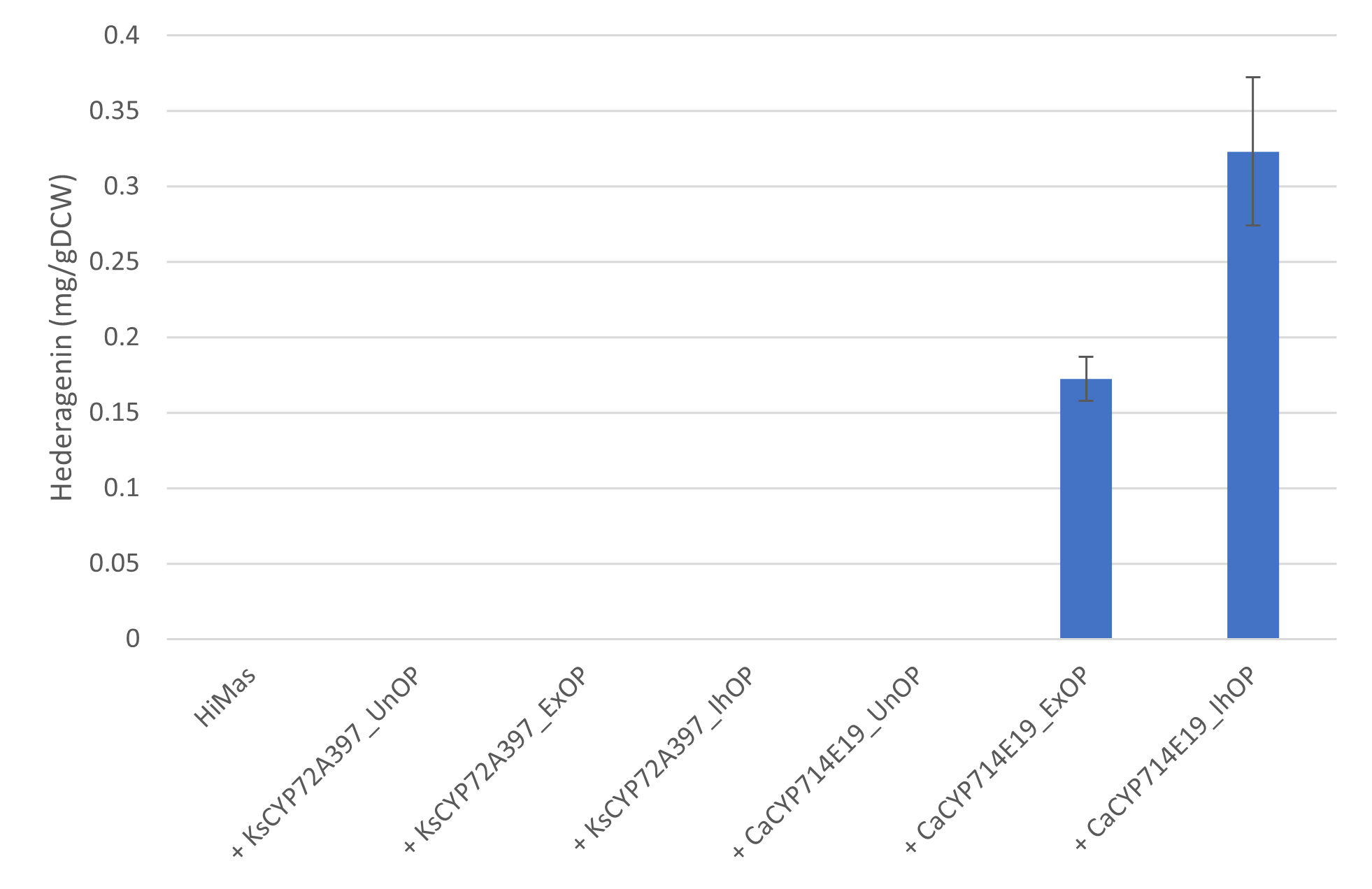


Supplementary Figure 5: Hederagenin production by HiMas-based strains expressing KsCYP72A397 or CaCYP714E1. UnOP, not codon optimized for Y. lipolytica. ExOp, codon optimized for Y. lipolytica using an external algorithm. IhOp, codon optimized for Y. lipolytica using an in-house algorithm. All titer averages and standard deviations are based three experimental replicates.


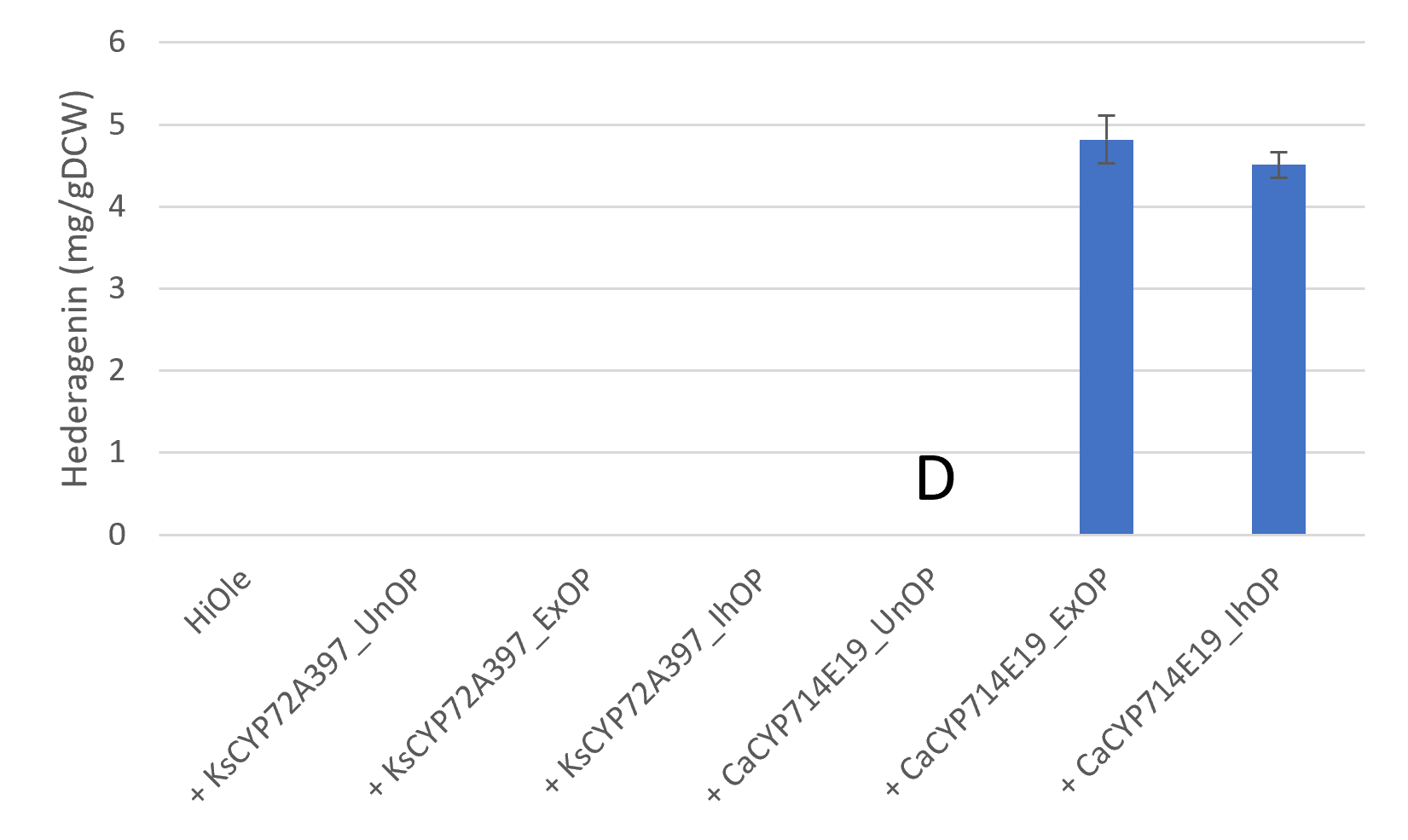


Figure 6: Hederagenin production by HiOle-based strains expressing KsCYP72A397 or CaCYP714E19. UnOP, not codon optimized for Y. lipolytica. ExOp, codon optimized for Y. lipolytica using an external algorithm. IhOp, codon optimized for Y. lipolytica using an in-house algorithm. All titer averages and standard deviations are based three experimental replicates. D, detected.


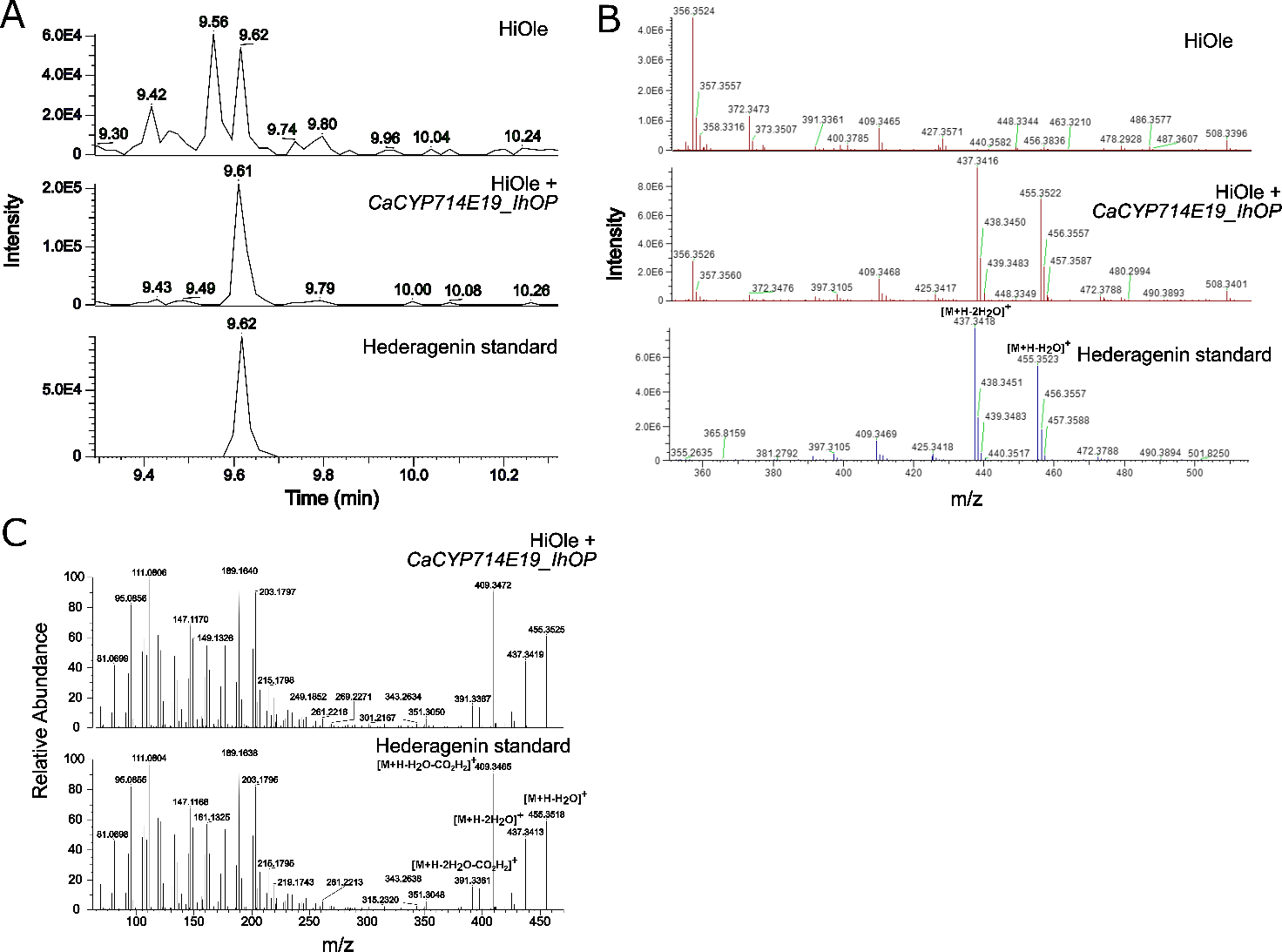


Figure 7: LC-MS analysis for the presence of hederagenin. **(A)** Extracted ion chromatograms of control (HiOle), HiOle expressing CaCYP714E19_IhOP, and an authentic hederagenin standard. **(B)** comparison of MS1 spectra from HiOle (RT: 9.62 min), HiOle expressing CaCYP714E19_IhOP (RT: 9.61 min), and the authentic hederagenin standard (RT: 9.62 min). Notably, the characteristic hederagenin single and double water loss ions are absent for the background peak (RT: 9.62 min) in the HiOle sample. **(C)** comparison of MS2 spectra from HiOle expressing CaCYP714E19_IhOP (RT: 9.62 min) and the authentic standard (RT: 9.62 min).


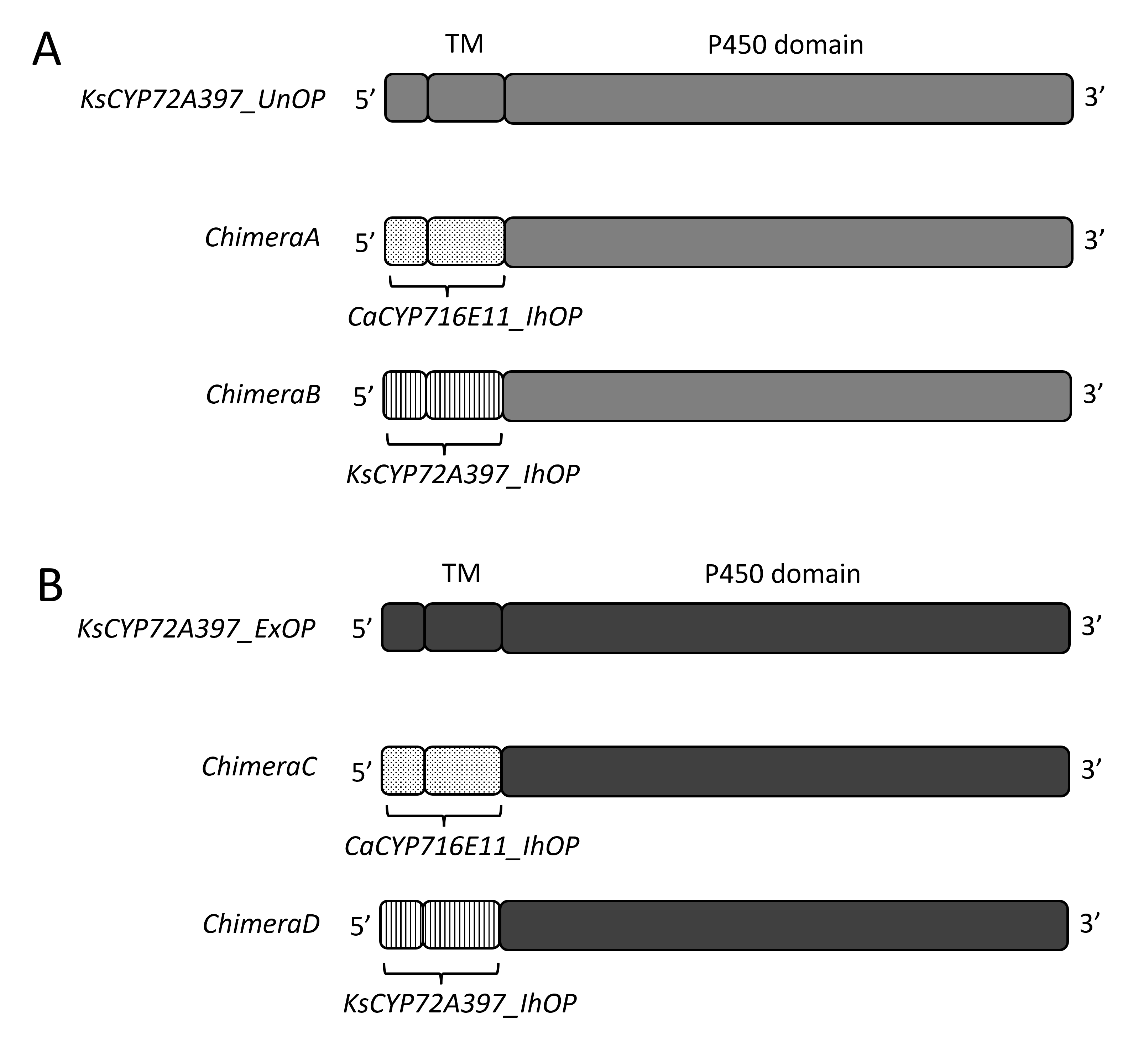


Supplementary Figure 8: **(A)** Overview of nucleotide sequences for KsCYP72A397_UnOP, ChimeraA, and ChimeraB. **(B)** Overview of nucleotide sequences for KsCYP72A397_ExOP, ChimeraC, and ChimeraD.


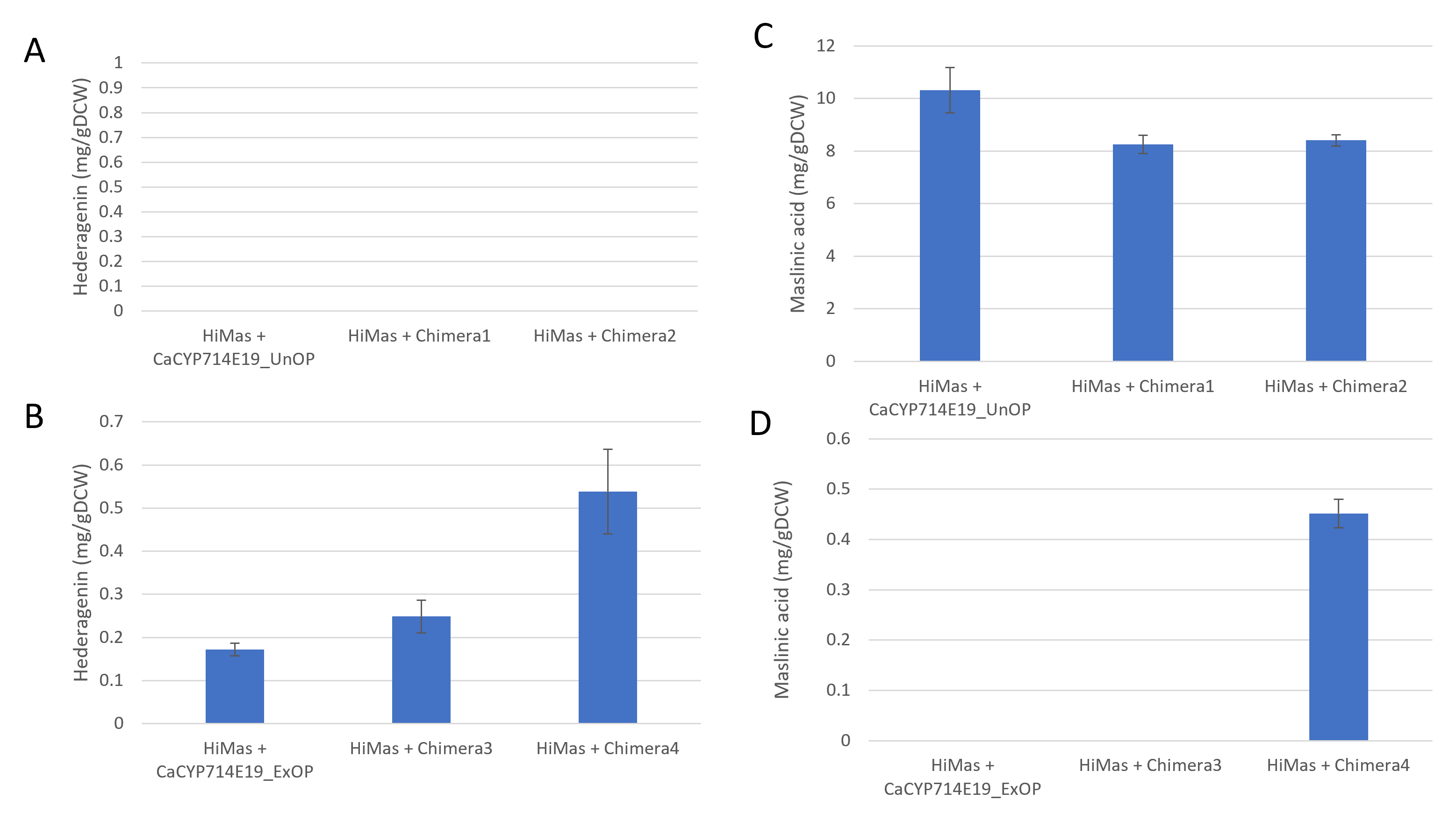


Supplementary Figure 9: Hederagenin and maslinic acid production by HiMas-based strains expressing Chimera1-4. **(A)** hedereganin production for Chimera1-2 expression in HiMas-background. **(B)** Hedereganin production for Chimera3-4 expression in HiMas-background. **(C)** Maslinic acid production for Chimera1-2 expression in HiMas-background. **(D)** Maslinic acid production for Chimera3-4 expression in HiMas-background. All titer averages and standard deviations are based three experimental replicates.

## Supplementary tables

**Supplementary table S1.** Strains used in this study.

| **Strain number** | **Genotype** | **Parental strain** | **Added vector elements/Reference** |
| --- | --- | --- | --- |
| ST9106  (C_30_-platform strain) | MATa ku70∆::PrTEF1-Cas9-TTef12::PrGPD-DsdA-TLip2 IntC_2-HMG1<-PrGPD-PrTefInt->ERG12 pERG7_50bp IntC_3-SeACS<-PrGPD-PrTefInt->YlACL1 IntD_1-IDI1<-PrGPD-PrTefInt->ERG20 IntE_1-SQE<-PrGPD-PrTefInt->SQS1 |  | (Arnesen et al., 2020) |
| ST9651 | ST9106 IntE_4-YlSQE<-GPD-tefInt->GgBAS | ST9106 | pCfB8861 (pHphM-YLgRNA2_IntE_4):pCfB9241 (IntE_4-YlSQE<-GPD-tefInt->GgBAS) |
| ST9653 | ST9106 IntE_4-tefInt->GgBAS-GSG-trYlSQE | ST9106 | pCfB8861 (pHphM-YLgRNA2_IntE_4):pCfB9243 (IntE_4-tefInt->GgBAS-GSG-trYlSQE) |
| ST9756  (HiOle) | ST9106 IntE_4-tefInt->GgBAS-GSG-trYlSQE IntF_3-AtATR2<-GPD-tefInt->MtCYP716A12 | ST9653 | pCfB9247 (IntF_3-AtATR2<-GPD-tefInt->MtCYP716A12) |
| ST10590  (HiMas) | ST9106 IntE_4-tefInt->GgBAS-GSG-trYlSQE IntF_3-AtATR2<-GP IntE_3_Tefin->CaCYP716C11_ExOP | ST9756 | pCfB10373 (IntE_3_Tefin->CaCYP716C11) + pCfB8860 (pHphM-YLgRNA2_IntE_3) |
| ST10978 (C_30,COOH_-platform strain) | ST9106 IntF_3-AtATR2<-GPD-tefInt->MtCYP716A12 | ST9106 | pBP8003 (pNat-YLgRNA4-IntF_3):pCfB9247 (IntF_3-AtATR2<-GPD-tefInt->MtCYP716A12) |
| ST10986  (HiUrs) | ST9106 IntF_3-AtATR2<-GPD-tefInt->MtCYP716A12 IntE_3_YlSQE<-GPD-tefint->MdOSC1m | ST10978 | pCfB6637 (pNat-YLgRNA3_IntE_3):pCfB10565 (IntE_3_YlSQE<-GPD-tefint->MdOSC1m) |
| ST10987 | ST9106 IntF_3-AtATR2<-GPD-tefInt->MtCYP716A12 IntE_3_trYlSQE<-GPD-tefint->MdOSC1m | ST10978 | pCfB6637 (pNat-YLgRNA3_IntE_3):pCfB10566 (IntE_3_trYlSQE<-GPD-tefint->MdOSC1m) |
| ST10988 | ST9106 IntF_3-AtATR2<-GPD-tefInt->MtCYP716A12 IntE_3_tefint->MdOSC1m-GSG-trYlSQE | ST10978 | pCfB6637 (pNat-YLgRNA3_IntE_3):pCfB10567 (IntE_3_tefint->MdOSC1m-GSG-trYlSQE) |
| ST11041 | ST9106 IntE_4-tefInt->GgBAS-GSG-trYlSQE IntF_3-AtATR2<-GPD-tefInt->MtCYP716A12 IntE_2_Nat_KsCYP72A397_ExOP | ST9756 | pCfB10629 (IntE_2_Nat_KsCYP72A397_ExOP) |
| ST11042 | ST9106 IntE_4-tefInt->GgBAS-GSG-trYlSQE IntF_3-AtATR2<-GPD-tefInt->MtCYP716A12 IntE_2_Nat_CaCYP714E19_IhOP | ST9756 | pCfB10630 (IntE_2_Nat_CaCYP714E19_IhOP) |
| ST11043 | ST9106 IntE_4-tefInt->GgBAS-GSG-trYlSQE IntF_3-AtATR2<-GPD-tefInt->MtCYP716A12 IntE_2_Nat_CaCYP714E19_UnOP | ST9756 | pCfB10632 (IntE_2_Nat_CaCYP714E19_UnOP) |
| ST11044 | ST9106 IntE_4-tefInt->GgBAS-GSG-trYlSQE IntF_3-AtATR2<-GPD-tefInt->MtCYP716A12 IntE_2_Nat_KsCYP72A397_UnOP | ST9756 | pCfB10633 (IntE_2_Nat_KsCYP72A397_UnOP) |
| ST11045 | ST9106 IntE_4-tefInt->GgBAS-GSG-trYlSQE IntF_3-AtATR2<-GP IntE_3_Tefin->CaCYP716C11_ExOP IntE_2_Nat_KsCYP72A397_ExOP | ST10590 | pCfB10629 (IntE_2_Nat_KsCYP72A397_ExOP) |
| ST11046 | ST9106 IntE_4-tefInt->GgBAS-GSG-trYlSQE IntF_3-AtATR2<-GP IntE_3_Tefin->CaCYP716C11_ExOP IntE_2_Nat_CaCYP714E19_IhOP | ST10590 | pCfB10630 (IntE_2_Nat_CaCYP714E19_IhOP) |
| ST11047 | ST9106 IntE_4-tefInt->GgBAS-GSG-trYlSQE IntF_3-AtATR2<-GP IntE_3_Tefin->CaCYP716C11_ExOP IntE_2_Nat_CaCYP714E19_UnOP | ST10590 | pCfB10632 (IntE_2_Nat_CaCYP714E19_UnOP) |
| ST11048 | ST9106 IntE_4-tefInt->GgBAS-GSG-trYlSQE IntF_3-AtATR2<-GP IntE_3_Tefin->CaCYP716C11_ExOP IntE_2_Nat_KsCYP72A397_UnOP | ST10590 | pCfB10633 (IntE_2_Nat_KsCYP72A397_UnOP) |
| ST11253 | ST9106 IntE_4-tefInt->GgBAS-GSG-trYlSQE IntF_3-AtATR2<-GPD-tefInt->MtCYP716A12 IntE_2_nat_Tefint->KsCYP72A397_IhOP | ST9756 | pCfB10710 (IntE_2_nat_Tefint->KsCYP72A397_IhOP) |
| ST11254 | ST9106 IntE_4-tefInt->GgBAS-GSG-trYlSQE IntF_3-AtATR2<-GP IntE_3_Tefin->CaCYP716C11_ExOP IntE_2_nat_Tefint->KsCYP72A397_IhOP | ST10590 | pCfB10710 (IntE_2_nat_Tefint->KsCYP72A397_IhOP) |
| ST11255 | ST9106 IntE_4-tefInt->GgBAS-GSG-trYlSQE IntF_3-AtATR2<-GP IntE_3_Tefin->CaCYP716C11_ExOP pIntE-2-Nat-tefint->Chimeric_A397_CaCYP714E19_ExOP | ST10590 | pCfB10749 (pIntE-2-Nat-tefint->Chimeric_A397_CaCYP714E19_ExOP) |
| ST11256 | ST9106 IntE_4-tefInt->GgBAS-GSG-trYlSQE IntF_3-AtATR2<-GP IntE_3_Tefin->CaCYP716C11_ExOP pIntE-2-Nat-tefint->Chimeric_A397_CaCYP714E19_UnOP | ST10590 | pCfB10750 (pIntE-2-Nat-tefint->Chimeric_A397_CaCYP714E19_UnOP) |
| ST11257 | ST9106 IntE_4-tefInt->GgBAS-GSG-trYlSQE IntF_3-AtATR2<-GP IntE_3_Tefin->CaCYP716C11_ExOP pIntE-2-Nat-tefint->Chimeric_A397_KsCYP72A397_ExOP | ST10590 | pCfB10751 (pIntE-2-Nat-tefint->Chimeric_A397_KsCYP72A397_ExOP) |
| ST11258 | ST9106 IntE_4-tefInt->GgBAS-GSG-trYlSQE IntF_3-AtATR2<-GP IntE_3_Tefin->CaCYP716C11_ExOP pIntE-2-Nat-tefint->Chimeric_A397_KsCYP72A397_UnOP | ST10590 | pCfB10752 (pIntE-2-Nat-tefint->Chimeric_A397_KsCYP72A397_UnOP) |
| ST11259 | ST9106 IntE_4-tefInt->GgBAS-GSG-trYlSQE IntF_3-AtATR2<-GP IntE_3_Tefin->CaCYP716C11_ExOP pIntE-2-Nat-tefint->Chimeric_E19_CaCYP714E19_ExOP | ST10590 | pCfB10753 (pIntE-2-Nat-tefint->Chimeric_E19_CaCYP714E19_ExOP) |
| ST11260 | ST9106 IntE_4-tefInt->GgBAS-GSG-trYlSQE IntF_3-AtATR2<-GP IntE_3_Tefin->CaCYP716C11_ExOP pIntE-2-Nat-tefint->Chimeric_E19_CaCYP714E19_UnOP | ST10590 | pCfB10754 (pIntE-2-Nat-tefint->Chimeric_E19_CaCYP714E19_UnOP) |
| ST11261 | ST9106 IntE_4-tefInt->GgBAS-GSG-trYlSQE IntF_3-AtATR2<-GP IntE_3_Tefin->CaCYP716C11_ExOP pIntE-2-Nat-tefint->Chimeric_E19_KsCYP72A397_ExOP | ST10590 | pCfB10755 (pIntE-2-Nat-tefint->Chimeric_E19_KsCYP72A397_ExOP) |
| ST11262 | ST9106 IntE_4-tefInt->GgBAS-GSG-trYlSQE IntF_3-AtATR2<-GP IntE_3_Tefin->CaCYP716C11_ExOP pIntE-2-Nat-tefint->Chimeric_E19_KsCYP72A397_UnOP | ST10590 | pCfB10756 (pIntE-2-Nat-tefint->Chimeric_E19_KsCYP72A397_UnOP) |
| ST11283 | ST9106 IntE_4-tefInt->GgBAS-GSG-trYlSQE IntF_3-AtATR2<-GPD-tefInt->MtCYP716A12 IntE_2_Nat_CaCYP714E19_ExOP | ST9756 | pCfB10628 (IntE_2_Nat_CaCYP714E19_ExOP) |
| ST11284 | ST9106 IntE_4-tefInt->GgBAS-GSG-trYlSQE IntF_3-AtATR2<-GP IntE_3_Tefin->CaCYP716C11_ExOP IntE_2_Nat_CaCYP714E19_ExOP | ST10590 | pCfB10628 (IntE_2_Nat_CaCYP714E19_ExOP) |
| ST11288 | ST9106 IntE_4-YlSQE<-GPD-tefInt->GgBAS IntF_3-AtATR2<-GPD-tefInt->MtCYP716A12 | ST9651 | pBP8003 (pNat-YLgRNA4-IntF_3):pCfB9247 (IntF_3-AtATR2<-GPD-tefInt->MtCYP716A12) |
| ST11290 | ST9106 IntF_3-AtATR2<-GPD-tefInt->MtCYP716A12 IntE_3_YlSQE<-GPD-tefint->MdOSC1m IntE_4-CaCYP716C11_ExOP<-GPD-tefInt->CaCYP714E19_IhOP | ST10986 | pCfB8861 (pHphM-YLgRNA2_IntE_4):pCfB10738 (IntE_4-CaCYP716C11_ExOP<-GPD-tefInt->CaCYP714E19_IhOP) |
| ST11295 | ST9106 IntF_3-AtATR2<-GPD-tefInt->MtCYP716A12 IntE_3_YlSQE<-GPD-tefint->MdOSC1m IntE_4-CaCYP716C11_ExOP<-GPD-tefInt->CaCYP714E19_IhOP pIntE-2-Nat-tefint->CaCYP716E41_IhOP | ST11290 | pCfB10740 (pIntE-2-Nat-tefint->CaCYP716E41_IhOP) |

**Supplementary table S2.** Plasmids used in this study.

| **Plasmid** | **Parent plasmid** | **Biobricks/Reference** |
| --- | --- | --- |
| pCfB4779 (pIntE_2-Nat-TPex20-TLip2) |  | (Holkenbrink et al., 2018) |
| pCfB6563 (pIntC-2-Nat-YlCyb5<-PrExp-PrGPD->AtATR2) |  | (Kildegaard et al., 2021) |
| pCfB6679 (pIntE_4-TPex20-TLip2) |  | (Holkenbrink et al., 2018) |
| pCfB6681 (pIntE_3-TPex20-TLip2) |  | (Holkenbrink et al., 2018) |
| pBP8009 (pIntF_3-TPex20-TLip2) |  | Lab collection, a kind gift from BioPhero APS. |
| pCfB8831 (IntE_1-SQE<-PrGPD-PrTefInt->SQS1) |  | (Arnesen et al., 2020) |
| pCfB9241 (IntE_4-YlSQE<-GPD-tefInt->GgBAS) | pCfB6679 (pIntE_4-TPex20-TLip2) | BB3865 (<-PrGDP_Tefint->):BB4218 (GgBAS):BB4217 (YlSQE) |
| pCfB9243 (IntE_4-tefInt->GgBAS-GSG-trYlSQE) | pCfB6679 (pIntE_4-TPex20-TLip2) | BB3879 (Tefint->):BB4224 (GgBAS_fusion):BB4226 (YlSQE_fusion) |
| pCfB9247 (IntF_3-AtATR2<-GPD-tefInt->MtCYP716A12) | pBP8009 (pIntF_3-TPex20-TLip2) | BB3865 (<-PrGDP_Tefint->):BB4222 (AtATR2):BB4221 (MtCYP716A12) |
| pCfB9253 (IntE_3-AtATR2<-GPD-tefInt-> KsCYP72A397) | pCfB6681 (pIntE_3-TPex20-TLip2) | BB3865 (<-PrGDP_Tefint->):BB4222 (AtATR2):BB4223 (KsCYP72A397) |
| pCfB9680 (pIntE_5-TPex20-TLip2) |  | Lab collection, a kind gift from BioPhero APS. |
| pCfB10372 (IntE_3_Tefin->CaCYP714E19) | pCfB6681 (pIntE_3-TPex20-TLip2) | BB3879 (Tefint->):BB5045 (CaCYP716C11(Tefin)) |
| pCfB10373 (IntE_3_Tefin->CaCYP716C11) | pCfB6681 (pIntE_3-TPex20-TLip2) | BB3879 (Tefint->):BB5046 (CaCYP714E19(Tefin)) |
| pCfB10562 (IntE_5-Tefint->KsCYP72A397_IhOP) | pCfB9680 (pIntE_5-TPex20-TLip2) | BB3879 (Tefint->):BB5163 (KsCYP72A397_IhOP) |
| pCfB10565 (IntE_3_YlSQE<-GPD-tefint->MdOSC1m) | pCfB6681 (pIntE_3-TPex20-TLip2) | BB5166 (YlSQE<-GPD-tefint->):BB5168 (MdOSC1m) |
| pCfB10566 (IntE_3_trYlSQE<-GPD-tefint->MdOSC1m) | pCfB6681 (pIntE_3-TPex20-TLip2) | BB5167 (trYlSQE<-GPD-tefint->):BB5168 (MdOSC1m) |
| pCfB10567 (IntE_3_tefint->MdOSC1m-GSG-trYlSQE) | pCfB6681 (pIntE_3-TPex20-TLip2) | BB3879 (Tefint->):BB5169 (MdOSC1m_for_fusion):BB5170 (YlSQE_for_fusion) |
| pCfB10628 (IntE_2_Nat_CaCYP714E19_ExOP) | pCfB4779 (pIntE_2-Nat-TPex20-TLip2) | BB3879 (Tefint->):BB5160 (CaCYP714E19_ExOP) |
| pCfB10629 (IntE_2_Nat_KsCYP72A397_ExOP) | pCfB4779 (pIntE_2-Nat-TPex20-TLip2) | BB5161 (Tefint->KsCYP72A397_ExOP) |
| pCfB10630 (IntE_2_Nat_CaCYP714E19_IhOP) | pCfB4779 (pIntE_2-Nat-TPex20-TLip2) | BB3879 (Tefint->):BB5162 (CaCYP714E19_IhOP) |
| pCfB10632 (IntE_2_Nat_CaCYP714E19_UnOP) | pCfB4779 (pIntE_2-Nat-TPex20-TLip2) | BB3879 (Tefint->):BB5165 (CaCYP714E19_UnOP) |
| pCfB10633 (IntE_2_Nat_KsCYP72A397_UnOP) | pCfB4779 (pIntE_2-Nat-TPex20-TLip2) | BB3879 (Tefint->):BB5164 (KsCYP72A397_UnOP) |
| pCfB10710 (IntE_2_nat_Tefint->KsCYP72A397_IhOP) |  | BB5263 (IntE_2_nat_backbone_pcrUSER):BB5264 (Tefint->KsCYP72A397_Jondah_opt) |
| pCfB10738 (IntE_4-CaCYP716C11_ExOP<-GPD-tefInt->CaCYP714E19_IhOP) |  | BB5273 (IntE_4_backbone_user):BB5280 (CaCYP716C11_ExOP_gpd):BB5279 (CaCYP714E19_IhOP_new) |
| pCfB10740 (pIntE-2-Nat-tefint->CaCYP716E41_IhOP) |  | BB5263 (IntE_2_nat_backbone_pcrUSER):BB5265 (Tefint_pcrUSER):BB5281 (CaCYP716E41_IhOP) |
| pCfB10749 (pIntE-2-Nat-tefint->Chimeric_A397_CaCYP714E19_ExOP) |  | BB5294 (IntE_2_Nat_A397_JDopt_leader_backbone):BB5299 (A397_CaCYP714E19_ExOP) |
| pCfB10750 (pIntE-2-Nat-tefint->Chimeric_A397_CaCYP714E19_UnOP) |  | BB5294 (IntE_2_Nat_A397_JDopt_leader_backbone):BB5300 (A397_CaCYP714E19_UnOP) |
| pCfB10751 (pIntE-2-Nat-tefint->Chimeric_A397_KsCYP72A397_ExOP) |  | BB5294 (IntE_2_Nat_A397_JDopt_leader_backbone):BB5301 (A397_KsCYP72A397_ExOP) |
| pCfB10752 (pIntE-2-Nat-tefint->Chimeric_A397_KsCYP72A397_UnOP) |  | BB5294 (IntE_2_Nat_A397_JDopt_leader_backbone):BB5302 (A397_KsCYP72A397_UnOP) |
| pCfB10753 (pIntE-2-Nat-tefint->Chimeric_E19_CaCYP714E19_ExOP) |  | BB5293 (IntE_2_Nat_E19_JDopt_leader_backbone):BB5295 (E19_CaCYP714E19_ExOP) |
| pCfB10754 (pIntE-2-Nat-tefint->Chimeric_E19_CaCYP714E19_UnOP) |  | BB5293 (IntE_2_Nat_E19_JDopt_leader_backbone):BB5296 (E19_CaCYP714E19_UnOP) |
| pCfB10755 (pIntE-2-Nat-tefint->Chimeric_E19_KsCYP72A397_ExOP) |  | BB5293 (IntE_2_Nat_E19_JDopt_leader_backbone):BB5297 (E19_KsCYP72A397_ExOP) |
| pCfB10756 (pIntE-2-Nat-tefint->Chimeric_E19_KsCYP72A397_UnOP) |  | BB5293 (IntE_2_Nat_E19_JDopt_leader_backbone):BB5298 (E19_KsCYP72A397_UnOP) |

**Supplementary table S3.** Synthetic genes used in this study.

| **Synthetic genes** | **Codon optimization algorithm** |
| --- | --- |
| pCfB9232 (GgBAS_ExOP) | (Swainston et al., 2014) |
| pCfB9233 (KsCYP72A397_ExOP) | (Swainston et al., 2014) |
| pCfB9235 (MtCYP716A12_ExOP) | (Swainston et al., 2014) |
| pCfB10549 (CaCYP714E19_IhOP) | In-house algorithm |
| pCfB10550 (KsCYP72A397_IhOP) | In-house algorithm |
| pCfB10551 (KsCYP72A397_UnOP) | No codon optimization |
| pCfB10552 (CaCYP714E19_UnOP) | No codon optimization |
| pCfB10553 (MdOSC1m_ExOP) | (Fath et al., 2011) |
| pCfB10737 (CaCYP716E41_IhOP) | In-house algorithm |
| pCfB11115 (CaCYP716C11_ExOP) | (Fath et al., 2011) |
| pCfB11116 (CaCYP714E19_ExOP) | (Fath et al., 2011) |

**Supplementary table S4.** Biobricks used in this study.

| **Biobrick** | **Template/reference** | **Forward primer** | **Reverse**  **primer** |
| --- | --- | --- | --- |
| BB3865 (<-PrGDP_Tefint->) | (Arnesen et al., 2020) |  |  |
| BB3879 (Tefint->) | (Arnesen et al., 2020) |  |  |
| BB4218 (GgBAS) | pCfB9232 (GgBAS_ExOP) | 25334 | 25335 |
| BB4221 (MtCYP716A12) | pCfB9235 (MtCYP716A12_ExOP) | 25340 | 25341 |
| BB4222 (AtATR2) | pCfB6563 (pIntC-2-Nat-YlCyb5<-PrExp-PrGPD->AtATR2) | 25342 | 25343 |
| BB4223 (KsCYP72A397) | pCfB9233 (KsCYP72A397_ExOP) | 25346 | 25347 |
| BB4224 (GgBAS_fusion) | pCfB9232 (GgBAS_ExOP) | 25334 | 25348 |
| BB4226 (YlSQE_fusion) | pCfB8831 (IntE_1-SQE<-PrGPD-PrTefInt->SQS1) | 25351 | 25352 |
| BB5045 (CaCYP716C11(Tefin)) | pCfB11115 (CaCYP716C11_ExOP) | 27957 | 27958 |
| BB5046 (CaCYP714E19(Tefin)) | pCfB11116 (CaCYP714E19_ExOP) | 27955 | 27956 |
| BB5160 (CaCYP714E19_ExOP) | pCfB10372 (IntE_3_Tefin->CaCYP714E19) | 28761 | 27956 |
| BB5161 (Tefint->KsCYP72A397_ExOP) | pCfB9253 (IntE_3-AtATR2<-GPD-tefInt-> KsCYP72A397) | 23847 | 25347 |
| BB5162 (CaCYP714E19_IhOP) | pCfB10549 (CaCYP714E19_IhOP) | 28762 | 28763 |
| BB5163 (KsCYP72A397_IhOP) | pCfB10550 (KsCYP72A397_IhOP) | 28764 | 28765 |
| BB5164 (KsCYP72A397_UnOP) | pCfB10551 (KsCYP72A397_UnOP) | 28766 | 28767 |
| BB5165 (CaCYP714E19_UnOP) | pCfB10552 (CaCYP714E19_UnOP) | 28768 | 28769 |
| BB5166 (YlSQE<-GPD-tefint->) | pCfB9241 (IntE_4-YlSQE<-GPD-tefInt->GgBAS) | 27640 | 18214 |
| BB5168 (MdOSC1m) | pCfB10553 (MdOSC1m_ExOP) | 28770 | 28771 |
| BB5169 (MdOSC1m_for_fusion) | pCfB10553 (MdOSC1m_ExOP) | 28770 | 28772 |
| BB5170 (YlSQE_for_fusion) | pCfB9241 (IntE_4-YlSQE<-GPD-tefInt->GgBAS) | 28773 | 25352 |
| BB5263 (IntE_2_nat_backbone_pcrUSER) | pCfB4779 (pIntE_2-Nat-TPex20-TLip2) | 28980 | 28981 |
| BB5264 (Tefint->KsCYP72A397_Jondah_opt) | pCfB10562 (IntE_5-Tefint->KsCYP72A397_IhOP) | 28983 | 28765 |
| BB5265 (Tefint_pcrUSER) | pCfB9247 (IntF_3-AtATR2<-GPD-tefInt->MtCYP716A12) | 28983 | 18214 |
| BB5273 (IntE_4_backbone_user) | pCfB6679 (pIntE_4-TPex20-TLip2) | 28980 | 28981 |
| BB5279 (CaCYP714E19_IhOP_new) | pCfB10630 (IntE_2_Nat_CaCYP714E19_IhOP) | 28762 | 29001 |
| BB5280 (CaCYP716C11_ExOP_gpd) | pCfB10373 (IntE_3_Tefin->CaCYP716C11) | 29002 | 29003 |
| BB5281 (CaCYP716E41_IhOP) | pCfB10737 (CaCYP716E41_IhOP) | 29004 | 29005 |
| BB5293 (IntE_2_Nat_E19_JDopt_leader_backbone) | pCfB10630 (IntE_2_Nat_CaCYP714E19_IhOP) | 28981 | 28982 |
| BB5294 (IntE_2_Nat_A397_JDopt_leader_backbone) | pCfB10710 (IntE_2_nat_Tefint->KsCYP72A397_IhOP) | 28981 | 28997 |
| BB5295 (E19_CaCYP714E19_ExOP) | pCfB10628 (IntE_2_Nat_CaCYP714E19_ExOP) | 28984 | 28992 |
| BB5296 (E19_CaCYP714E19_UnOP) | pCfB10632 (IntE_2_Nat_CaCYP714E19_UnOP) | 28985 | 28993 |
| BB5297 (E19_KsCYP72A397_ExOP) | pCfB10629 (IntE_2_Nat_KsCYP72A397_ExOP) | 28986 | 28994 |
| BB5298 (E19_KsCYP72A397_UnOP) | pCfB10633 (IntE_2_Nat_KsCYP72A397_UnOP) | 28987 | 28995 |
| BB5299 (A397_CaCYP714E19_ExOP) | pCfB10628 (IntE_2_Nat_CaCYP714E19_ExOP) | 28988 | 28992 |
| BB5300 (A397_CaCYP714E19_UnOP) | pCfB10632 (IntE_2_Nat_CaCYP714E19_UnOP) | 28989 | 28993 |
| BB5301 (A397_KsCYP72A397_ExOP) | pCfB10629 (IntE_2_Nat_KsCYP72A397_ExOP) | 28990 | 28994 |
| BB5302 (A397_KsCYP72A397_UnOP) | pCfB10633 (IntE_2_Nat_KsCYP72A397_UnOP) | 28991 | 28995 |

**Supplementary table S5.** Primers used in this study.

| **Primer number** | **Sequence** |
| --- | --- |
| 18214 | AGTACTGCAAAAAGUGCTG |
| 23847 | cgtgcgaUAGAGACCGGGTTGGCGGCGCAT |
| 25334 | ACTTTTTGCAGTACUAACCGCAGTGGCGACTCAAGATCGCTGA |
| 25335 | CACGCGAUTTAAGTGAGGCACACAGGAGTGG |
| 25340 | ACTTTTTGCAGTACUAACCGCAGGAGCCCAACTTCTACCTCTC |
| 25341 | CACGCGAUTCAGGCCTTGTGGGGGTACA |
| 25342 | ATCTGTCAUGCCACAATGTCCTCTTCGTCGTCCTC |
| 25343 | CGTGCGAUTCACCACACATCTCGCAGGT |
| 25346 | ACTTTTTGCAGTACUAACCGCAGGACGGTGTGGTTGTCACCTA |
| 25347 | CACGCGAUTTACAGCTTGTGCAGAATCAGG |
| 25348 | AGGCACACAGGAGUGGAAGGGA |
| 25351 | ACTCCTGTGTGCCUCACTGGCTCCGGCAATCAGGGCAGAAAGGTTC |
| 25352 | CACGCGAUCTAAGTCAGCTCGCTCCAA |
| 27640 | CGTGCGAUCTAAGTCAGCTCGCTCCAAATG |
| 27955 | ACTTTTTGCAGTACUAACCGCAGATGGAGCTGGAGAACTACTCTTCCGA |
| 27956 | CACGCGAUTTACAGCTTCTTGACCATCAGTTTGACACC |
| 27957 | ACTTTTTGCAGTACUAACCGCAGATGGACCTGTTTCTCCCCCTCGTC |
| 27958 | CACGCGAUTTAGTGGGGGTGCAGTCGGATG |
| 28761 | ACTTTTTGCAGTACUAACCGCAGGAACTCGAGAACTACTCTTCTGA |
| 28762 | ACTTTTTGCAGTACUAACCGCAGGAGCTCGAGAACTACTCCTCCG |
| 28763 | CACGCGAUTTAGAGCTTCTTGACCATGAGCT |
| 28764 | ACTTTTTGCAGTACUAACCGCAGGACGGTGTCGTCGTCACCTA |
| 28765 | CACGCGAUTTAGAGCTTGTGGAGGATGAGGT |
| 28766 | ACTTTTTGCAGTACUAACCGCAGGATGGAGTGGTAGTCACATACAC |
| 28767 | CACGCGAUTTAAAGCTTGTGCAAAATCAAGT |
| 28768 | ACTTTTTGCAGTACUAACCGCAGGAGTTGGAAAATTATAGTAGTGATATTG |
| 28769 | CACGCGAUTCACAGCTTCTTCACCATGAGTT |
| 28770 | ACTTTTTGCAGTACUAACCGCAGTGGAAGATCAAGTTCGGCGA |
| 28771 | CACGCGAUTTAGGCGATCTTCTTAATGGGCAG |
| 28772 | AGCCGGCGATCTUCTTAATGGGCAGAGACACCAGA |
| 28773 | AAGATCGCCGGCUCCGGCaatcagggcagaaaggttcttg |
| 28980 | attccgttgaagUgtggatggggaagtgagtgc |
| 28981 | atcgcgtgcaUtccttctgttcggaatcaacct |
| 28982 | AGACGAAGGCUCGGCAGAAACCCA |
| 28983 | acttcaacggaaUgcgtgcgatAGAGACCGGGTTGGCGGC |
| 28984 | AGCCTTCGTCUGGGAGCCCAAGCGACTGCGA |
| 28985 | AGCCTTCGTCUGGGAACCAAAGAGGCTTAGATCT |
| 28986 | AGCCTTCGTCUGGCCACGAAAGCTGGAGGAATC |
| 28987 | AGCCTTCGTCUGGCCACGAAAGCTAGAGGAGTC |
| 28988 | ACTGGGTCTGGGUCTCCGAGCCCAAGCGACTGCGA |
| 28989 | ACTGGGTCTGGGUCTCCGAACCAAAGAGGCTTAGATCTT |
| 28990 | ACTGGGTCTGGGUCTCCCCACGAAAGCTGGAGGAATC |
| 28991 | ACTGGGTCTGGGUCTCCCCACGAAAGCTAGAGGAGTCC |
| 28992 | atgcacgcgaUTTACAGCTTCTTCACCATCAGC |
| 28993 | atgcacgcgaUTCACAGCTTCTTCACCATGAGT |
| 28994 | atgcacgcgaUTTACAGCTTGTGCAGAATCAGG |
| 28995 | atgcacgcgaUTTAAAGCTTGTGCAAAATCAAGTTA |
| 28997 | ACCCAGACCCAGUTGAGGACCTTC |
| 29001 | atgcacgcgaUTTAGAGCTTCTTGACCATGAGC |
| 29002 | ATCTGTCAUGCCACAATGGACCTGTTCCTGCCTCT |
| 29003 | acttcaacggaaUgcgtgcgatTTAGTGAGGGTGCAGTCGG |
| 29004 | ACTTTTTGCAGTACUAACCGCAGTCCCTCTTCTCCGACGTCG |
| 29005 | atgcacgcgaUTTAGTTCTTGTGGGGGACGAGTC |

## Synthetic genes

*GgBAS_ExOP*

ATGTGGCGACTCAAGATCGCTGAGGGAGGAAAGGATCCTTACATTTATTCTACCAACAACTTCGTCGGTCGACAGACCTGGGAGTACGATCCCGACGGCGGTACCCCCGAAGAGCGAGCTCAGGTTGACGCCGCTCGGCTGCACTTCTATAACAACCGATTTCAGGTTAAGCCCTGCGGAGATCTGCTCTGGCGATTCCAGATCCTGCGAGAGAACAACTTCAAACAGACCATCGCCAGTGTGAAGATCGGAGACGGAGAAGAGATCACGTACGAGAAGGCCACCACCGCCGTGCGACGAGCTGCGCACCACCTGTCCGCTCTGCAAACTTCTGACGGCCACTGGCCCGCCCAGATCGCAGGACCCCTGTTCTTCTTGCCACCTCTGGTGTTTTGCATGTACATCACCGGCCACCTCGACTCCGTGTTCCCCGAGGAGTATCGCAAGGAGATCTTGCGATACATCTACTATCACCAAAACGAAGATGGAGGCTGGGGTCTCCACATCGAGGGACATTCTACCATGTTCTGCACCGCCCTTAACTACATCTGCATGCGAATCCTCGGTGAGGGCCCTGACGGAGGCCAGGATAACGCCTGCGCCCGTGCTCGAAAGTGGATCCATGATCACGGTGGTGTCACTCACATTCCTTCTTGGGGAAAGACCTGGCTGTCTATTCTGGGAGTCTTCGATTGGTGCGGATCGAACCCCATGCCTCCCGAATTCTGGATCTTGCCTTCTTTCCTGCCCATGCACCCTGCCAAGATGTGGTGCTACTGCCGACTGGTGTACATGCCCATGTCCTACCTGTACGGTAAGCGATTCGTGGGACCAATCACTCCCCTGATTCTGCAGCTTCGAGAGGAGCTGTTTACTGAACCCTACGAGAAAGTCAACTGGAAGAAGGCCCGGCACCAGTGCGCCAAGGAGGACCTGTACTACCCTCACCCCCTGCTCCAGGACCTCATTTGGGACTCCCTTTACCTGTTTACCGAGCCTCTCCTGACCCGATGGCCCTTCAACAAGCTGGTGAGAGAAAAGGCCCTCCAGGTGACCATGAAGCACATCCACTACGAGGACGAGACTTCCCGATACATTACCATTGGATGTGTTGAGAAGGTCCTGTGTATGCTGGCTTGTTGGGTCGAAGATCCCAACGGTGACGCCTTCAAGAAGCACCTTGCCAGAGTCCCCGACTACCTTTGGGTGTCTGAGGACGGCATGACCATGCAGTCTTTCGGCTCCCAGGAATGGGACGCTGGTTTTGCCGTGCAAGCCCTTCTCGCTACCAACCTGGTCGAGGAGATCGCACCCACCCTGGCCAAGGGACACGACTTCATCAAGAAGTCCCAGGTCAGAGACAACCCTTCCGGAGACTTCAAGTCTATGTACCGCCACATCTCGAAGGGTTCTTGGACCTTCTCCGACCAGGACCACGGCTGGCAGGTGAGCGATTGCACTGCTGAGGGTCTGAAGTGCTGTCTGCTGTTGTCCATGCTGCCCCCTGAAATTGTTGGTGAGAAGATGGAACCGGAACGGCTGTATGACTCTGTGAACGTCCTGCTGTCCCTGCAGTCCAAGAAAGGTGGTCTGTCTGCCTGGGAGCCCGCTGGAGCACAAGAGTGGCTCGAGCTGCTGAACCCCACCGAGTTCTTTGCTGACATCGTCGTCGAGCACGAGTACGTCGAATGCACTGGTTCCGCTATTCAGGCCCTTGTGCTGTTTAAGAAGCTGTACCCCGGTCACCGAAAGAAGGAAATCGAAAACTTCATCGCCAACGCTGTTCGATTTCTGGAGGACACCCAAACCGCCGACGGATCCTGGTACGGTAACTGGGGCGTGTGCTTCACCTACGGCTCTTGGTTCGCCCTGGGTGGACTCGCCGCTGCCGGTAAGACTTTCGCCAACTGTGCTGCCATTCGAAAGGCCGTGAAGTTTCTGCTTACTACCCAGCGAGAGGACGGTGGTTGGGGTGAGTCCTACCTTTCTTCTCCTAAGAAGATCTACGTCCCTCTGGAGGGTTCTCGATCCAACGTCGTCCACACCGCTTGGGCCCTTATGGGTCTGATTCACGCCGGACAGGCCGAACGGGATCCTGCCCCGCTCCACCGAGCCGCCAAGCTCATCATCAACTCTCAGCTGGAGGAGGGTGACTGGCCTCAGCAGGAAATCACCGGGGTCTTTATGAAGAACTGCATGCTCCACTATCCCATGTACCGAGACATCTACCCTATGTGGGCTCTTGCTGAATACCGACGACGAGTCCCTCTCCCTTCCACTCCTGTGTGCCTCACTTAA

*MdOSC1^m^_ExOP*

ATGTGGAAGATCAAGTTCGGCGAGGGCGCTACTGACCCCATGCTGTTCTCTACCAACAACTTCCACGGCCGACAGACCTGGGAGTTCGACCCCGACGCCGGCACCGAAGAGGAACGAGCCGAGGTCGAGGCCGCTCGAGAGCACTTCTACCAGAACCGATTCAAGGTGCAGCCCTCTTCTGACCTGCTGTGGCGATTCCAGATCCTGCGAGAGAAGAACTTCAAGCAAGAGATCCCTCCTGTGCGAGTCGGCGAAGGCGAGGACATCACCTACGACCAGGCCACCGCCGCCTTCCGACGAGCCGCCACCTTCTGGAACGCCCTGCAGTCTCCCCACGGACACTGGCCCGCTGAGAACGCTGGCCCCAACTTCTACTTCCCTCCTCTGGTGATGGCCGCCTACATTCCCGGCTACCTGAACGTGATCTTCTCTGCCGAGCACAAGAAGGAAATCCTGCGATACACCTACAACCACCAGAACGAGGACGGCGGCTGGGGCCTGCACATTGCTGGCCCCTCTATGATGTTCACCACCTGTCTGAACTACTGCATGATGCGAATCCTCGGCGACGGCCCCGACGGCGGACGAGACAACGCCTGCGCTCGAGCCCGAAAGTGGATCCTGGACCGAGGCGGAGCCTACTACTCTGCCTCTTGGGGCAAGACCTGGATGGCCATTCTGGGCGTGTACGACTGGGAGGGCTCTAACCCCATGCCTCCTGAGTTCTGGACCGGCTCTACCCTGCTGCACTTTCACCCCTCCAAGATGTTCTGCTACTGCCGACTGACCTACCTGCCTATGTCTTACTTCTACGCTACCCGATTCGTGGGCCCCATCACACCCCTGGTCGAGGAACTGCGACAAGAGATCTACTGCGAGTCTTACAACGAGATCAACTGGCCCAAGGTGCGACACTGGTGCGCCACCGAGGACAACTACTACCCTCACGGCCGAGTGCAGCGATTCATGTGGGACGGCTTCTACAACATCGTCGAGCCCCTGCTGAAGAGATGGCCCTTCAAGAAGATCCGAGATAACGCCATCCAGTTCACCATCGACCAGATCCACTACGAGGACGAGAACTCTCGGTACATCACCATCGGCTGTGTCGAGAAGGCCCTGATGATGCTGGCCTGCTGGGCCGAAGATCCTTCTGGCGAGGCTTTCAAGAAGCACTTGCCCCGAGTCACCGACTACATCTGGCTCGGCGAGGACGGAATCAAGATGCAGTCTTTCGGCTCTCAGTCTTGGGACTGCGCCCTGGTGATTCAGGCCCTGCTGGCCGGAAACCTGAACGCCGAGATGGGCCCCACTCTGAAGAAGGCCCACGAGTTTCTGAAGATCTCTCAGGTGCGAATCAACACCTCTGGCGACTACCTGTCTCACTTCCGACACATCTCTAAGGGCGCCTGGACCTTCTCCGACCGAGATCACGGCTGGCAGGTTTCTGACTGCACCGCCGAGGCTCTCCGATGCTGCTGCATCTTCGCCAACATGTCTCCCGAGGTGGTGGGCGAGCCCATGGAAGCCGAGTGTATGTACGACGCCGTGAACGTCATCATGTCTCTGCAGTCGCCCAACGGCGGCGTGTCTGCCTGGGAGCCCACCGGCGCTCCCAAGTGGCTCGAGTGGCTGAACCCCGTCGAGTTTCTCGAGGACCTGGTGATCGAGTACGAGTATATCGAGTGCACCTCTTCGTCTATCCAGGCTCTGACCCTGTTCCGAAAGCTGTACCCCGGCCACCGACGAAAGGAAATCAACAACTTTATCACCCGAGCCGCTGACTACATCGAGGACATTCAGTACCCCGACGGATCTTGGTACGGCAACTGGGGCATCTGCTTCGTGTACGGCACCTGGTTCGCCATCAAGGGACTCGAGGCTGCCGGCCGAACCTACAACAACTGCGAGGCCGTGCGAAAGGGCGTCGACTTCCTGCTCAAGACCCAGCGAGCTGACGGTGGATGGGGCGAGCACTACACCTCTTGCACCAACAAGAAGTACACCGCTCAGGACTCCACCAACCTGGTGCAGACCGCTCTGGGCCTGATGGGACTGATTCACGGACGACAGGCCGAGCGAGATCCCACTCCTATCCACCGAGCCGCCGCTGTGCTGATGAACGGCCAGCTGGACGACGGCGACTTCCCTCAGCAAGAGCTGATGGGCGTGTTCATGCGAAACGCCATGCTGCACTACGCCGCCTACCGAAACATCTTCCCTCTGTGGGCCCTGGGCGAGTACCGAACTCTGGTGTCTCTGCCCATTAAGAAGATCGCCTAA

*KsCYP72A397_UnOP*

ATGGATGGAGTGGTAGTCACATACACAAAAATTGCAGCGGCGGTGGCTGTGGCTGTTGTTGTTGTTGGATGGGCGTGGAAGGTGTTGAATTGGGTGTGGGTGAGTCCACGAAAGCTAGAGGAGTCCCTCAGAAAACAAGGATTCCGTGGAAATTCGTACCGTCTGTTTTATGGGGACCTGAAAGAAAGCTCGGAAATGACGAGGAAAGCTAAGTTGAAGCCCATCAATCTCTCTGATGATCCTGTGCTGCGGGTCAGGCCCTTTATCCATCAAACCGTTAAGAAATACGGTAAGAGTTCCTTTATATGGATTGGACCTACGCCAAGGGTGCAAATAATGGACCCTGAAATTATAAAAGAAATTATGGTTAAGAGTTACAAATTCAACAAGCCCAAGAGGAACCCACTAGTCAAGTTGTTTGCTGATGGGCTCGCAAACCATGAAGGGGAGCTGTGGGCCAAACATAGAAAACTACTTAATCCGGCTTTCCATCTAGAGAGGTTGAAGTGTATGCTACCAGCCATGTATTTTAGCTGTATAGAGATGGTGAGCAAATGGGATAAAATGATTTCGAAGGATGGGTCACGTGAGTTGGACGTGTGGCCTTTTTTGCAAAGATTAACAAGCGACGTCATCTCACACACGGCATTTGGAAGTAGCTACGAAGAGGGAAATATAGTATTTGAACTTCAAACAGAACAAGCTGAGCTTGTAATGAAGACCCTACAATCAGTTTACATTCCCGGATGGAGTTATCTACCAACTAAGAGGAACAGGAAGATGAAGGAAATTGACCGAAAAACACAATCCTGTTTGATGAACATTATCAATAAAAAGACGAAGGCCATGCAGGCAGGAGAAGGTAGCACTGACGACATACTAGGGATACTACTGGAATCCAATTTAAAGGAACAGCTTGGACAAGGGAAAAAAAATGTTGGAATGAGTATTCAAGAGGTTATGGGAGAGTGCAAGCAATTCTATTTTGCTGGGCAGGAGACCACCTCCGGTTTGCTTGTCTGGACTATGGTTTTATTGAGCATTCACCCGAATTGGCAAGCACGCGCTAGAGAAGAGGTTCTTCAACAATTTGGAAATGCAAAACCGGATTTTGATAATCTAAATCACCTCAAAATCGTTACCATGATTTTATACGAAGTTTTAAGGTTATATCCACCTGTTGATACGCTGTTCCGAAGGGTTGACCAGGAGACTACATTAGGAGATATAACCTTACCAGCAGGAGTTCAAATCTCGTTACCAATCATGATACTTCACCATGACCAAAATATTTGGGGCGATGATGCTAAGGAGTTTAATCCAGAAAGATTTTCTGAGGGAGTGTCAAAGGCAACAAAAAATCAGGTCGTGTTTTTCCCATTTGGCTGGGGACCGAGGATCTGCATTGGACAAAACTTTGCATTGTTGGAAGCAAAATTAGCTCTTGCTATCATCTTACAACGCTTCTCCTTTGAGCTCTCGCCATCCTATACCCATGCCCCGACCACTGTCCTGACTGTTCAACCACAACATGGAGCTAACTTGATTTTGCACAAGCTTTAA

*KsCYP72A397_ExOP*

ATGGACGGTGTGGTTGTCACCTACACCAAGATCGCCGCCGCTGTGGCCGTCGCCGTTGTTGTCGTGGGATGGGCTTGGAAGGTTCTCAACTGGGTTTGGGTCTCCCCACGAAAGCTGGAGGAATCGCTGCGAAAGCAGGGCTTTCGAGGAAACTCCTACCGACTTTTCTACGGTGACCTCAAGGAGTCTTCTGAGATGACTCGAAAGGCCAAGCTGAAGCCCATTAACCTGTCCGACGACCCCGTCCTGCGGGTTCGACCCTTCATCCACCAGACCGTTAAGAAGTACGGAAAGTCTTCTTTTATTTGGATCGGCCCCACTCCTCGAGTCCAGATTATGGATCCTGAGATCATCAAGGAGATTATGGTGAAGTCTTACAAGTTTAACAAGCCCAAGCGAAACCCACTGGTGAAGCTGTTCGCCGACGGACTCGCCAACCACGAGGGCGAGCTGTGGGCTAAGCATCGAAAGCTGCTGAACCCTGCTTTTCACCTGGAGCGACTGAAGTGTATGCTTCCCGCTATGTACTTCTCTTGTATCGAGATGGTTTCCAAGTGGGACAAGATGATTTCTAAGGACGGATCTCGTGAGCTGGATGTCTGGCCTTTCCTTCAGCGACTCACTTCCGACGTCATCTCCCACACCGCTTTCGGATCTTCCTACGAGGAGGGTAACATCGTGTTCGAGCTGCAGACCGAACAAGCCGAGCTCGTCATGAAGACCCTGCAATCTGTCTACATTCCCGGCTGGTCCTACCTGCCCACCAAGCGCAATCGAAAGATGAAAGAGATCGACCGAAAAACTCAGTCTTGTTTAATGAACATCATCAACAAGAAGACCAAGGCTATGCAGGCCGGCGAGGGCTCCACCGACGATATCTTGGGCATTCTGCTGGAGTCTAACCTGAAGGAGCAACTTGGTCAGGGTAAGAAGAACGTCGGTATGTCCATTCAGGAGGTCATGGGCGAGTGCAAGCAGTTCTACTTTGCCGGTCAGGAGACTACCTCCGGTCTGCTGGTTTGGACTATGGTCCTGCTCTCCATCCACCCCAACTGGCAGGCCCGTGCCCGAGAGGAGGTCCTTCAGCAGTTCGGTAACGCCAAGCCCGATTTCGACAACCTGAACCATCTGAAGATTGTCACCATGATCCTCTACGAGGTCCTCCGACTGTATCCCCCTGTGGACACACTGTTCCGACGAGTTGATCAGGAAACCACTCTGGGCGACATCACTCTGCCTGCTGGTGTTCAGATCTCTCTGCCCATCATGATCCTGCATCACGACCAGAACATCTGGGGTGACGACGCCAAGGAGTTCAACCCCGAACGATTCTCCGAGGGAGTCAGCAAGGCTACCAAGAACCAGGTTGTCTTCTTCCCCTTCGGTTGGGGTCCGCGAATTTGCATTGGCCAGAACTTCGCCCTGCTCGAAGCCAAACTTGCTCTGGCCATTATTCTGCAGCGATTCTCCTTCGAGCTGTCCCCCTCCTACACCCACGCTCCCACCACTGTCCTTACCGTCCAGCCTCAGCACGGTGCCAACCTGATTCTGCACAAGCTGTAA

*KsCYP72A397_IhOP*

ATGGACGGTGTCGTCGTCACCTACACCAAGATCGCCGCTGCCGTCGCCGTTGCCGTCGTCGTCGTCGGTTGGGCCTGGAAGGTCCTCAACTGGGTCTGGGTCTCCCCCCGAAAGCTCGAGGAGTCCCTCCGAAAGCAGGGTTTCCGAGGTAACTCCTACCGACTCTTCTACGGTGACCTCAAGGAGTCCTCCGAGATGACCCGAAAGGCCAAGCTCAAGCCCATCAACCTCTCCGACGACCCCGTCCTCCGAGTCCGACCCTTCATCCACCAGACCGTCAAGAAGTACGGTAAGTCCTCCTTCATCTGGATCGGTCCCACCCCCCGAGTCCAGATCATGGACCCCGAGATCATCAAGGAGATCATGGTCAAGTCCTACAAGTTCAACAAGCCCAAGCGAAACCCCCTCGTCAAGCTCTTCGCCGACGGTCTCGCCAACCACGAGGGTGAGCTCTGGGCCAAGCACCGAAAGCTCCTCAACCCCGCCTTCCACCTCGAGCGACTCAAGTGCATGCTCCCCGCCATGTACTTCTCCTGCATCGAGATGGTCTCCAAGTGGGACAAGATGATCTCCAAGGACGGTTCCCGAGAGCTCGACGTCTGGCCCTTCCTCCAGCGACTCACCTCCGACGTCATCTCCCACACCGCCTTCGGTTCCTCCTACGAGGAGGGTAACATCGTCTTCGAGCTCCAGACCGAGCAGGCCGAGCTCGTCATGAAGACCCTCCAGTCCGTCTACATCCCCGGTTGGTCCTACCTCCCCACCAAGCGAAACCGAAAGATGAAGGAGATCGACCGAAAGACCCAGTCCTGCCTCATGAACATCATCAACAAGAAGACCAAGGCCATGCAGGCCGGTGAGGGTTCCACCGACGACATCCTCGGTATCCTCCTCGAGTCCAACCTCAAGGAGCAGCTCGGTCAGGGTAAGAAGAACGTCGGTATGTCCATCCAGGAGGTCATGGGTGAGTGCAAGCAGTTCTACTTCGCCGGTCAGGAGACCACCTCCGGTCTCCTCGTCTGGACCATGGTCCTCCTCTCCATCCACCCCAACTGGCAGGCCCGAGCCCGAGAGGAGGTCCTCCAGCAGTTCGGTAACGCCAAGCCCGACTTCGACAACCTCAACCACCTCAAGATCGTCACCATGATCCTCTACGAGGTCCTCCGACTCTACCCCCCCGTCGACACCCTCTTCCGACGAGTCGACCAGGAGACCACCCTCGGTGACATCACCCTCCCCGCCGGTGTCCAGATCTCCCTCCCCATCATGATCCTCCACCACGACCAGAACATCTGGGGTGACGACGCCAAGGAGTTCAACCCCGAGCGATTCTCCGAGGGTGTCTCCAAGGCCACCAAGAACCAGGTCGTCTTCTTCCCCTTCGGTTGGGGTCCCCGAATCTGCATCGGTCAGAACTTCGCCCTCCTCGAGGCCAAGCTCGCCCTCGCCATCATCCTCCAGCGATTCTCCTTCGAGCTCTCCCCCTCCTACACCCACGCCCCCACCACCGTCCTCACCGTCCAGCCCCAGCACGGTGCCAACCTCATCCTCCACAAGCTCTAA

*CaCYP716C11_ExOP*

ATGGACCTGTTCCTGCCTCTGGTGTTCCTGTCTGTGATCCTGATCGTGCTGATCTTCAAGCCCCGATCTGACGGCGACAAGAAGCTGCCTCCTGGCTCTTTCGGCTGGCCCATCATGGGCGAGACTATCGAGTTCCTGTTCGGACACCCCAAGGAATTCGTGGACAAGCGAATGAAGAAGTACTCTCCCGACATCTTCAAGTCTAACATCCTGGGCGAAAAGACCGCCATCATCTGTGGCCCCGAGGGACACAAGTTTCTGTTCTCTAACGAAGAGAAGTTCTTCACCGTGTTCCGACCTCATCCTATCCAGCGACTGTTCCGATCTTACAACAACAAGTCTGCTCCCGATCCTCCTCCTTCTGGCGCCGGATCTAAGGACGACGTGAAGTCTATCAAGCAGCCCGGCTTCTTTAAGCCCGAGGCTCTGTCTCGATTCATCGGCGTGATCGAGGCCACCATCCAGCAGCATCTGCGAGCCCACTGGGAAGGCAAGGACACCGTCGAGGCTTACCCTCTGTCTAAGTCTCTGACCCTGACTCTGTCTTGCCGATTCTTCCTGGGCATCGACAACCCCGAGCGAATCGCCCGACTGGTGCACATGTTCGACGACATCACCCTGGGCATGCACTCTATCATCTCTAACGTGCCCGGAACCGTGTTCTACCGAGCCAAGAACGCCGCTGCCGCCGTGCGAAAGGAACTGCTGTGCGTGATCAAGGAAAAGAAGCAAGAGATGGCCGCTGGCAAGAAGGCCCAGGACGTTCTGTCTCACATGATTTCTTTCTCGGACCCCTCTACCGGCAAGTTCATGCCCGAGCTGGAAGTGGCCGACAAGATGATGGGACTGATCACCGCCGGCTACTCTACCGTGGCTACCTCTATGGCCTTCCTGATGAAGTTCGTGGGCGAGTCTCCCGCCATCTACAACAAGATCCGAGCCGAGCAGATCGAGCTGGCCGAGTCTAAGAACCCCGGCGAGCCCCTGACCTGGGTCGACATCCAGAAGCTGAAGTACTCCTGGCAGGCCATGTGTGAGACTATGCGACTCGTGCCTCCTCTGCAGGGCACCTTCCGAGAGGTGATCAACGAGTTCACCTACGCCGGATACACCGTGCCTAAGGGCTGGAAGGTGTACTGGACCGTGTCCACCGTGCACATGAACCCCAAGTACTTCCCCAACCCTGAGAAGTTTGACCCCTCTCGATACGAAGAGGGCAAGATCTCTACCCCTTACACCTACGTGCCCTTCGGCGGAGGACCCCGAATGTGCCCCGGCAAGGAATACGCCCGAATCGCCGTGCTGACCTTCCTGCACCATGTGGTGCGAAAGTACAAGTGGGAAGTGCTGTTCCCCGACGAGAAGGTGATCGGCGACATGATGCCCGCACCTGAGAAGGGACTGCCCATCCGACTGCACCCTCACTAA

*CaCYP714E19_UnOP*

ATGGAGTTGGAAAATTATAGTAGTGATATTGTGTTGAAAGTGATGATATCCATAGGTGTGATTGGAGTATTGGGACTGGTAATGGGGTTTTGTAGAGCATTTGTTTGGGAACCAAAGAGGCTTAGATCTTTGTTGAAGAAACAAGGGATTGATGGACCAGAGCCAAAGATTGTTGTTGGGAATTTACTGGATATGAAGAATGCTTCTAAGAACAAACCTCCTGTTGATTCTTCTTCTGGCATCTCTCACAACGGTTTATCTTTTCTTTTTCCTATTTTCAAGAAATGGAAAGATCAATATGGTCCATTATTCGTATTTTCACTTGCAAACATGAACGTACTGTTTGTACACAAACCTGATGTGGTGAAAGAAATAACCACACACACATCCTTGGACTTGGGAAAACCATCCTATCAGGAAAAAAACCTCGGCCCCTTGCTTGGTCGAGGCATATTGACCTCCAATGGTGAGTCTTGGGCTCATCAAAGGAAAATTCTTGCTCCTGAACTGTACATGGACAAAGTTAAGGGGATGTTACATATAATTACAGAATCTGCAATGACGTTGATAGACACATGGAAAACACAAATAGATGCTCAAGGGGGTGTTGCAGATATAACAGTCGATGAGCATATGAGAAATTTCTCCGGGAGTGTTATATCAAAAGCTTGTTTTGGTGGAAGTTATGCCAAAGGAGAAAAAATCTTCACAAAGCTCAGAGATCTCCAGGAGCTTTACACTAAAACGGGTTTACCATTTCAAATTCCGGGAATGAGACATTTACCAACTAAGAAAAACAGAAAATCTTGGGCGTTGCAGAAGGAAATTGGAGATTTGATTCTAAACGTGGTACATGAAAGGAAAGAAGTTGGATATGAAAAGGACTTATTGCAGATGGTACTGGAAGGTGCTGAACAGAGTAAATTCAGCCAAGAAGAAACAAACAGGTTCATTGTTGATAATTGTAAAAACATCTACCTAGCTGGATATGAAACTACTGCAGTTTCTTTGACATGGACACTGATGCTTTTGGCTTCAAACCCCGAATGGCAAACTCGCGTTCGCGACGAGGCACTCGAGGTTTGTAAGGGCCAAATTCCAACTAACGACATGCTTCTCAAGATGAAACAGCTAACAATGGTCATTTATGAGTCACTGCGGTTGTATTCTCCGGTGCCAGTGATTTCGAGGGAAGCCTTTAAAGACTTGAAATTTGCAGATGTAAATGTCCCAAAGGGTGTGAATGTATGGGGTGTAATATATTCATTGCACACAGACCCTGAAGTATGGGGGCCAGATTCTTTCGAGTTCAACCCCAACAGATTTGAAAATGGAACAAGAAACGCATGCAAGTATCCACAATACTATTGCCCATTCGGAGTCGGACCTCGGGTATGTCTCGGACAGAATTTGGCAATAGTTGAGCTGAAAGCACTAGTGTCTCTCCTCGTCTCTAACTTCTCTTTCGCTCTATCCTCGGACTACGTGCACTCCCCTGTAATCAAGCTGGTAATAGAGCCACAACATGGAGTTAAACTCATGGTGAAGAAGCTGTGA

*CaCYP714E19_ExOP*

ATGGAACTCGAGAACTACTCTTCTGACATCGTGCTGAAGGTGATGATCTCTATCGGCGTGATCGGAGTGCTGGGCCTCGTGATGGGCTTCTGCCGAGCCTTCGTGTGGGAGCCCAAGCGACTGCGATCTCTGCTGAAGAAGCAGGGCATCGACGGCCCCGAGCCTAAGATCGTGGTGGGCAACCTGCTGGACATGAAGAACGCCTCTAAGAACAAGCCTCCTGTGGACTCTTCTTCTGGCATCTCTCACAACGGCCTGTCTTTTCTGTTCCCCATCTTCAAGAAGTGGAAGGACCAGTACGGACCCCTGTTCGTGTTCTCTCTGGCCAACATGAACGTGCTGTTCGTGCACAAGCCCGACGTGGTGAAGGAAATTACTACCCACACCTCTCTGGACCTGGGCAAGCCCTCTTACCAAGAGAAGAACCTGGGACCTCTGCTCGGACGAGGCATCCTGACCTCTAACGGCGAGTCTTGGGCCCACCAGCGAAAGATTCTGGCTCCCGAGCTGTACATGGACAAGGTGAAGGGCATGCTGCACATCATCACCGAGTCTGCTATGACCCTGATCGACACCTGGAAGACCCAGATCGACGCCCAAGGCGGCGTGGCCGACATCACCGTGGACGAGCACATGCGAAACTTCTCTGGCTCTGTGATCTCTAAGGCCTGCTTCGGCGGCTCTTACGCCAAGGGCGAGAAGATTTTCACCAAGCTGCGAGATCTGCAAGAACTGTACACCAAGACCGGACTGCCCTTTCAGATCCCCGGCATGCGACATCTGCCCACCAAGAAGAACCGAAAGTCCTGGGCTCTGCAGAAGGAAATCGGCGACCTGATCCTGAACGTGGTGCACGAGCGAAAGGAAGTGGGCTACGAGAAGGACCTGCTGCAGATGGTGCTCGAGGGCGCCGAGCAGTCTAAGTTCTCTCAAGAGGAAACTAACCGATTCATCGTGGACAACTGTAAGAACATCTACCTGGCCGGATACGAGACTACCGCCGTGTCTCTGACCTGGACTCTGATGCTGCTGGCTTCTAACCCCGAGTGGCAGACCCGAGTGCGAGATGAGGCCCTCGAGGTGTGCAAGGGACAGATCCCCACCAACGACATGCTGCTCAAGATGAAGCAGCTGACCATGGTGATCTACGAGTCTCTGCGACTGTACTCTCCCGTGCCTGTGATTTCTCGAGAGGCCTTCAAGGACCTGAAGTTCGCCGACGTGAACGTGCCCAAGGGCGTGAACGTCTGGGGCGTGATCTACTCTCTGCACACTGACCCCGAGGTCTGGGGACCCGACTCTTTCGAGTTCAACCCCAACCGATTCGAGAACGGCACCCGAAACGCCTGCAAGTACCCTCAGTACTACTGCCCCTTCGGCGTGGGTCCCCGAGTGTGCCTGGGCCAGAACCTGGCCATCGTCGAGCTGAAGGCCCTGGTGTCTCTGCTGGTGTCTAACTTCTCTTTCGCCCTGTCCTCTGACTACGTGCACTCTCCTGTGATCAAGCTGGTGATCGAGCCCCAGCACGGCGTGAAGCTGATGGTGAAGAAGCTGTAA

*CaCYP714E19_IhOP*

ATGGAGCTCGAGAACTACTCCTCCGACATCGTCCTCAAGGTCATGATCTCCATCGGTGTCATCGGTGTCCTCGGTCTCGTCATGGGTTTCTGCCGAGCCTTCGTCTGGGAGCCCAAGCGACTCCGATCCCTCCTCAAGAAGCAGGGTATCGACGGTCCCGAGCCCAAGATCGTCGTCGGTAACCTCCTCGACATGAAGAACGCCTCCAAGAACAAGCCCCCCGTCGACTCCTCCTCCGGTATCTCCCACAACGGTCTCTCCTTCCTCTTCCCCATCTTCAAGAAGTGGAAGGACCAGTACGGTCCCCTCTTCGTCTTCTCCCTCGCCAACATGAACGTCCTCTTCGTCCACAAGCCCGACGTCGTCAAGGAGATCACCACCCACACCTCCCTCGACCTCGGTAAGCCCTCCTACCAGGAGAAGAACCTCGGTCCCCTCCTCGGTCGAGGTATCCTCACCTCCAACGGTGAGTCCTGGGCCCACCAGCGAAAGATCCTCGCCCCCGAGCTCTACATGGACAAGGTCAAGGGTATGCTCCACATCATCACCGAGTCCGCCATGACCCTCATCGACACCTGGAAGACCCAGATCGACGCCCAGGGTGGTGTCGCCGACATCACCGTCGACGAGCACATGCGAAACTTCTCCGGTTCCGTCATCTCCAAGGCCTGCTTCGGTGGTTCCTACGCCAAGGGTGAGAAGATCTTCACCAAGCTCCGAGACCTCCAGGAGCTCTACACCAAGACCGGTCTCCCCTTCCAGATCCCCGGTATGCGACACCTCCCCACCAAGAAGAACCGAAAGTCCTGGGCCCTCCAGAAGGAGATCGGTGACCTCATCCTCAACGTCGTCCACGAGCGAAAGGAGGTCGGTTACGAGAAGGACCTCCTCCAGATGGTCCTCGAGGGTGCCGAGCAGTCCAAGTTCTCCCAGGAGGAGACCAACCGATTCATCGTCGACAACTGCAAGAACATCTACCTCGCCGGTTACGAGACCACCGCCGTCTCCCTCACCTGGACCCTCATGCTCCTCGCCTCCAACCCCGAGTGGCAGACCCGAGTCCGAGACGAGGCCCTCGAGGTCTGCAAGGGTCAGATCCCCACCAACGACATGCTCCTCAAGATGAAGCAGCTCACCATGGTCATCTACGAGTCCCTCCGACTCTACTCCCCCGTCCCCGTCATCTCCCGAGAGGCCTTCAAGGACCTCAAGTTCGCCGACGTCAACGTCCCCAAGGGTGTCAACGTCTGGGGTGTCATCTACTCCCTCCACACCGACCCCGAGGTCTGGGGTCCCGACTCCTTCGAGTTCAACCCCAACCGATTCGAGAACGGTACCCGAAACGCCTGCAAGTACCCCCAGTACTACTGCCCCTTCGGTGTCGGTCCCCGAGTCTGCCTCGGTCAGAACCTCGCCATCGTCGAGCTCAAGGCCCTCGTCTCCCTCCTCGTCTCCAACTTCTCCTTCGCCCTCTCCTCCGACTACGTCCACTCCCCCGTCATCAAGCTCGTCATCGAGCCCCAGCACGGTGTCAAGCTCATGGTCAAGAAGCTCTAA

*CaCYP716E41_IhOP*

ATGTCCCTCTTCTCCGACGTCGTCCTCCTCGTCATCTTCCCCATCATCCTCTCCCTCTTCTTCTACCGAAAGCTCAAGTCCTCCTCCTCCAAGACCGTCAACACCCCCCCTGGTTCCTCCGGTTGGCCCATCGTCGGTGAGTCCATCAAGTTCGGTTCCGCCGGTCCTCAGAAGTTCATCAAGGAGCGAATGGAGAAGTACTCCCCCGACGTCTTCCACACCTCCCTCCTCGGTGAGAAGCTCGCCGTCTTCTGCGGTGCCCAGGGTAACAAGTTCGTCTTCACCAACGAGACTACCGTCCTCACCTCCTGGTGGCCCCAGGCTATGAAGAAGGCCCTCATCTCCCCCGAGCTCTCCGAGAACGAGGCCAAGGAGATCCGAAAGTCCCAGAAGGGTTTCCTCCCCGACATCCTCAAGCCCGAGGCCCTTAAGCAGTACATCCCCGTCATGGACGCCATGGCCCGAGATCACGTCGCCTCCGAGTGGACCCCCCGATACGTCGTCAAGGTTTCCCCCCTCGCTATGAAGTACACCTTCGGTCTGGCCTGCCGACTCTTCATGAACGTCGTCGAGCCCGACCGAATCGACAAGCTCACCAAGCACTTCACCCGAGTCATCACCGGTTTCATGGCCGTCCCCATCGACCTCCCCGGTACCGCTTACAACTCCGCCATCAACGGTGGTAAGCTCGTCCGAGCCGAGCTCCTCAACATCATCTCCGCCCGACGAGAGGAGATCCGAGAGAACAAGGAGACTATCGGTCGAGACTTCCTCTCCAAGATGCTCCTCGTCACCGACGAGGACGGTGAGTTCATGACCGAGAAGGAGATCTGCAACAACATCATCGGTCTGCTCGTCGCCTCCTACGAGACTACCTCCACCGCCGTCACCTTCGTCCTCAAGCACCTCGCCGAGTACCCCCACATCTACGACAAGGTCTACGAGGAGCAGATGGAGATCAAGAAGTCCAAGAAGCCCGGTGACCTCCTCTGCTGGGAGGACGTCCAGAAGATGAAGTACACCTGGAACGTCGCCTGCGAGTCCCTCCGACTCGCCCCTCCTGGTCAGGGTGCCTTCCGAGAGGCCGCTACCGACTTCACCTACGCCGGTTTCACCATCCCCAAGGGTTGGAAGGTCTTCTGGACCGTCCACACCTCCCACCGAGATCCCAAGTACTTCCCCGACCCCGATAAGTTCGACCCCTCCCGATTCGAGGGTTCCGGTCCCGCTCCTTACTCCTACGTCCCCTTCGGTGGTGGTCCCCGAATGTGCCCCGGTAAGGAGTACGCCCGACTCGAGATCCTCGTCTTCATCTACAACGTCGTCACCAACTTCAAGCTCGAGAAGCTCTCCCCCAACGAGAAGGTCCTCTTCCGATCCTCCCTCGTCCTCACCGAGGGTCTGCCCGTCCGACTCGTCCCCCACAAGAACTAA

*AtATR2* (as described in (Kildegaard et al., 2021))

ATGTCCTCTTCGTCGTCCTCTTCTACCTCTATGATCGACCTGATGGCCGCCATCATCAAGGGCGAGCCCGTGATCGTGTCTGACCCCGCCAACGCCTCTGCCTACGAGTCTGTGGCCGCCGAGCTGTCCTCTATGCTGATCGAGAACCGACAGTTCGCCATGATCGTGACCACCTCTATCGCCGTGCTGATCGGCTGCATCGTGATGCTGGTGTGGCGACGATCTGGCTCTGGCAACTCTAAGCGAGTCGAGCCCCTGAAGCCCCTGGTGATCAAGCCCCGAGAGGAGGAGATCGACGACGGCCGAAAGAAGGTGACCATCTTCTTCGGCACCCAGACCGGCACTGCCGAGGGCTTCGCCAAGGCCCTGGGCGAGGAGGCCAAGGCCCGATACGAGAAGACCCGATTCAAGATCGTGGACCTGGACGACTACGCCGCCGACGACGACGAGTACGAGGAGAAGCTGAAGAAGGAGGACGTCGCCTTCTTCTTCCTGGCCACCTACGGCGACGGCGAGCCCACCGACAACGCCGCTCGATTCTACAAGTGGTTCACCGAGGGCAACGACCGAGGCGAGTGGCTGAAGAACCTGAAGTACGGCGTGTTCGGCCTGGGCAACCGACAGTACGAGCACTTCAACAAGGTGGCCAAGGTGGTGGACGACATCCTGGTCGAGCAGGGCGCTCAGCGACTGGTGCAGGTCGGCCTGGGCGACGACGACCAGTGCATCGAGGACGACTTCACCGCCTGGCGAGAGGCCCTGTGGCCCGAGCTGGACACCATCCTGCGAGAGGAGGGCGACACCGCCGTCGCCACCCCTTACACCGCCGCTGTGCTCGAGTACCGAGTGTCTATCCACGACTCTGAGGACGCCAAGTTCAACGACATCAACATGGCCAACGGCAACGGCTACACCGTGTTCGACGCCCAGCACCCCTACAAGGCCAACGTGGCCGTGAAGCGAGAGCTGCACACCCCCGAGTCTGACCGATCTTGCATCCACCTCGAGTTCGACATTGCCGGCTCTGGCCTGACCTACGAGACTGGCGACCACGTGGGCGTGCTGTGCGACAACCTGTCTGAGACTGTGGACGAGGCCCTGCGACTGCTGGACATGTCTCCCGACACCTACTTCTCGCTGCACGCCGAGAAGGAGGATGGAACCCCCATCTCTTCGTCGCTGCCCCCTCCCTTCCCCCCCTGCAACCTGCGAACCGCCCTGACCCGATACGCCTGCCTGCTGTCCTCGCCCAAGAAGTCTGCCCTGGTCGCCCTGGCCGCTCACGCTTCTGACCCCACCGAGGCCGAGCGACTGAAGCACCTGGCCTCTCCCGCCGGAAAGGACGAGTACTCTAAGTGGGTGGTCGAGTCTCAGCGATCTCTGCTCGAGGTGATGGCCGAGTTCCCCTCGGCCAAGCCCCCTCTGGGCGTGTTCTTCGCCGGCGTGGCTCCCCGACTGCAGCCCCGATTCTACTCTATCTCTTCGTCCCCCAAGATTGCCGAGACTCGAATCCACGTGACCTGCGCCCTGGTGTACGAGAAGATGCCCACCGGCCGAATCCACAAGGGCGTGTGCTCTACCTGGATGAAGAACGCCGTGCCCTACGAGAAGTCTGAGAACTGTTCTTCTGCCCCCATCTTCGTGCGACAGTCTAACTTCAAGCTGCCCTCTGACTCTAAGGTGCCCATCATCATGATCGGACCCGGCACCGGCCTGGCTCCCTTCAGAGGCTTCCTGCAGGAGCGACTGGCCCTGGTCGAGTCTGGCGTCGAGCTGGGCCCCTCTGTGCTGTTCTTCGGCTGCCGAAACCGACGAATGGACTTCATCTACGAGGAGGAGCTGCAGCGATTCGTCGAGTCTGGTGCCCTGGCTGAGCTGTCTGTGGCCTTCTCGCGAGAGGGACCCACCAAGGAGTACGTCCAGCACAAGATGATGGACAAGGCCTCTGACATCTGGAACATGATCTCTCAGGGCGCCTACCTGTACGTGTGCGGCGACGCCAAGGGCATGGCCCGAGATGTGCACCGATCTCTGCACACCATTGCCCAGGAGCAGGGCTCTATGGACTCTACCAAGGCCGAGGGATTCGTGAAGAACCTGCAGACCTCTGGCCGATACCTGCGAGATGTGTGGTGA

**References**

Arnesen, J.A., Kildegaard, K.R., Cernuda Pastor, M., Jayachandran, S., Kristensen, M., Borodina, I., 2020. *Yarrowia* *lipolytica* Strains Engineered for the Production of Terpenoids. Front. Bioeng. Biotechnol. 8, 1–14. https://doi.org/10.3389/fbioe.2020.00945

Fath, S., Bauer, A.P., Liss, M., Spriestersbach, A., Maertens, B., Hahn, P., Ludwig, C., Schäfer, F., Graf, M., Wagner, R., 2011. Multiparameter RNA and Codon Optimization: A Standardized Tool to Assess and Enhance Autologous Mammalian Gene Expression. PLoS One 6, 1–14. https://doi.org/10.1371/journal.pone.0017596

Holkenbrink, C., Dam, M.I., Kildegaard, K.R., Beder, J., Dahlin, J., Doménech Belda, D., Borodina, I., 2018. EasyCloneYALI: CRISPR/Cas9-Based Synthetic Toolbox for Engineering of the Yeast *Yarrowia* *lipolytica*. Biotechnol. J. 13, 1700543. https://doi.org/10.1002/biot.201700543

Kildegaard, K.R., Arnesen, J.A., Adiego-Pérez, B., Rago, D., Kristensen, M., Klitgaard, A.K., Hansen, E.H., Hansen, J., Borodina, I., 2021. Tailored biosynthesis of gibberellin plant hormones in yeast. Metab. Eng. 66, 1–11. https://doi.org/10.1016/j.ymben.2021.03.010

Swainston, N., Currin, A., Day, P.J., Kell, D.B., 2014. GeneGenie: Optimized oligomer design for directed evolution. Nucleic Acids Res. 42, 395–400. https://doi.org/10.1093/nar/gku336
